# Global Transcriptome Analysis Identifies a Diagnostic Signature for Early Disseminated Lyme Disease and Its Resolution

**DOI:** 10.1128/mBio.00047-20

**Published:** 2020-03-17

**Authors:** Mary M. Petzke, Konstantin Volyanskyy, Yong Mao, Byron Arevalo, Raphael Zohn, Johanna Quituisaca, Gary P. Wormser, Nevenka Dimitrova, Ira Schwartz

**Affiliations:** a Department of Microbiology and Immunology, School of Medicine, New York Medical College, Valhalla, New York, USA; b Phillips Research North America, Valhalla, New York, USA; c Division of Infectious Diseases, Department of Medicine, New York Medical College, Valhalla, New York, USA; McGovern Medical School

**Keywords:** *Borrelia burgdorferi*, Lyme disease, diagnostics, random forest, transcriptome

## Abstract

A bioinformatics approach was employed to identify transcriptome alterations in the peripheral blood mononuclear cells of well-characterized human subjects who were diagnosed with early disseminated Lyme disease (LD) based on stringent microbiological and clinical criteria. Transcriptomes were assessed at the time of presentation and also at approximately 1 month (early convalescence) and 6 months (late convalescence) after initiation of an appropriate antibiotic regimen. Comparative transcriptomics identified 335 transcripts, representing 233 unique genes, with significant alterations of at least 2-fold expression in acute- or convalescent-phase blood samples from LD subjects relative to healthy donors. Acute-phase blood samples from LD subjects had the largest number of differentially expressed transcripts (187 induced, 54 repressed). This transcriptional profile, which was dominated by interferon-regulated genes, was sustained during early convalescence. 6 months after antibiotic treatment the transcriptome of LD subjects was indistinguishable from that of healthy controls based on two separate methods of analysis. Return of the LD expression profile to levels found in control subjects was concordant with disease outcome; 82% of subjects with LD experienced at least one symptom at the baseline visit compared to 43% at the early convalescence time point and only a single patient (9%) at the 6-month convalescence time point. Using the random forest machine learning algorithm, we developed an efficient computational framework to identify sets of 20 classifier genes that discriminated LD from other bacterial and viral infections. These novel LD biomarkers not only differentiated subjects with acute disseminated LD from healthy controls with 96% accuracy but also distinguished between subjects with acute and resolved (late convalescent) disease with 97% accuracy.

## Abstract (precis)

Lyme disease (LD), caused by Borrelia burgdorferi, is the most common tick-borne infectious disease in the United States. We examined gene expression patterns in the blood of individuals with early disseminated LD at the time of diagnosis (acute) and also at approximately 1 month and 6 months following antibiotic treatment. A distinct acute LD profile was observed that was sustained during early convalescence (1 month) but returned to control levels 6 months after treatment. Using a computer learning algorithm, we identified sets of 20 classifier genes that discriminate LD from other bacterial and viral infections. In addition, these novel LD biomarkers are highly accurate in distinguishing patients with acute LD from healthy subjects and in discriminating between individuals with active and resolved infection. This computational approach offers the potential for more accurate diagnosis of early disseminated Lyme disease. It may also allow improved monitoring of treatment efficacy and disease resolution.

## INTRODUCTION

Lyme disease (LD), a multisystem inflammatory disorder caused by Borrelia burgdorferi, is the most common tick-borne infectious disease in the United States, with an average of >25,000 reported cases per year during the past decade and an estimated annual incidence possibly as high as 300,000 cases per year (1). Diagnosis of early infection is primarily based on recognition of the characteristic skin lesion, erythema migrans (EM) (2–4). Treatment with appropriate antibiotics at this stage of infection is generally effective at preventing the development of later clinical manifestations (5–7). If left untreated, however, extracutaneous clinical manifestations may develop that can include neurologic manifestations (e.g., facial palsy), arthritis, or carditis (8–10).

Currently, detection of antibodies to B. burgdorferi is the mainstay of laboratory diagnosis of LD (11–13). However, there are several limitations of serologic testing, including lack of sensitivity in patients with EM and the inability of these tests to assess treatment response or to distinguish active from resolved infection (11, 14, 15). Transcriptional profiling of an infected host holds promise as an alternative to serologic testing for rapid and accurate diagnosis of recent infection. In studies unrelated to LD, both common transcriptional activation programs and pathogen-specific alterations in gene expression have been identified (16, 17), and several studies have demonstrated that this approach can discriminate between specific microbial infections, as well as predict disease outcome (18–22). Importantly, gene expression profiles have been used to differentiate between active and resolved infection (23–26). This technology offers the promise of overcoming certain limitations of LD serologic testing.

Here, we report on transcriptional profiling of patients with early LD who had objective evidence of disseminated infection and were evaluated both before and after antibiotic therapy. The random forest machine learning algorithm was employed to identify classifier gene sets that discriminate LD from other microbial infections. These novel gene sets differentiated subjects with acute disseminated LD from healthy controls with 96% accuracy. Notably, subjects with acute infection were also discriminated from those with resolved (late convalescent) disease with 97% accuracy.

## RESULTS

### Characteristics of study subjects.

The study included blood samples from 39 subjects with disseminated LD and from 21 healthy controls (Table 1). Different numbers of samples were included in the three time points used for evaluation of the LD subjects due to the following: some subjects failed to return for both of the follow-up visits, the amount and/or quality of RNA obtained from some blood samples was insufficient for analysis, and 6-month blood samples were collected only during the final 2 years of the study. Subjects who presented with physician-diagnosed EM from late May through early October were enrolled in the study, and an EM skin biopsy was performed. Confirmation of disseminated LD consisted of multiple erythema migrans (MEM) and/or isolation of B. burgdorferi from blood. The only exception was a study subject who presented with facial palsy, a sign of disseminated infection, and who was seropositive by two-tier serologic testing. Serologic testing by a first-tier whole-cell sonicate enzyme-linked immunosorbent assay (ELISA) was conducted at each sample collection time. All EM subjects except one were either seropositive by ELISA at presentation or seroconverted during the course of the study. B. burgdorferi was cultivated from the blood of 29 subjects with EM (Table 1).

**TABLE 1:** Clinical characteristics of human subjects

Parameter	Lyme disease subjects	Healthy donors
Total no. of subjects	39	21
Gender, no. (%)		
Male	22 (56)	9 (43)
Female	17 (44)	12 (57)
Age, no. (%)		
<60 yr	28 (68)	16 (76)
≥60 yr	11 (28)	3 (14)
EM rash		
Median size, cm^2^ (range)	104 (11–1,440)	
Median duration, days (range)	5 (1–60)	
MEM, no. (%)	26 (67)	
No. (%) seroreactive^*a*^ for *B. burgdorferi*		
Initial visit	28/38 (74)	0/21 (0)
One-month return visit	33/35 (94)	
Six-month return visit	6/11 (55)^*b*^	
Skin culture for *B. burgdorferi*		
No. (%) positive	22 (56)	
No. (%) negative	9 (23)	
No. (%) contaminated	1 (3)	
No. (%) not done	6 (15)	21 (100)
Blood culture for *B. burgdorferi*		
No. (%) positive	29 (74)	
No. (%) negative	7 (18)	
No. (%) not done	3 (8)	21 (100)
Disseminated infection		
No. (%) with MEM and/or positive blood culture	38/39 (95)^*c*^	

a That is, the number of subjects seroreactive/number of subjects examined. Whole-cell sonicate ELISA was used for Lyme disease subjects, and IgG immunoblotting was used for healthy donors.

b Includes four equivocal results.

c The remaining patient had facial palsy from Lyme disease.

### *B. burgdorferi* infection elicits a distinct gene expression signature during acute disease and early convalescence that resolves by 6 months following treatment.

**FIG 1:**
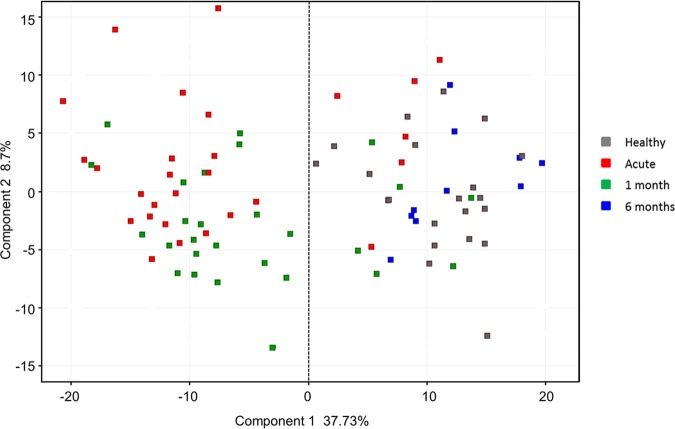
Principal-component analysis distinguishes subjects by disease state. Principal-component analysis of Lyme disease patients at three time points and healthy controls based on 335 differentially expressed transcripts (DETs).

To characterize the host response to B. burgdorferi infection, we compared gene expression in PBMCs from subjects with acute disseminated LD (*n* = 28), early convalescent LD (1 month; *n* = 27), and late convalescent LD (6 months; *n* = 10) with PBMCs from healthy donors (*n* = 21) using whole-genome oligonucleotide arrays. Principal-component analysis was performed using all samples. Figure 1 shows that the first principal component (*x* axis) accounts for 37.7% of the variability in the data and, with few exceptions, clearly separates the healthy donor and late convalescent LD blood samples from the acute and early convalescent LD blood samples. No further separation of samples within each of these groups occurs when the second (*y* axis) or third (*z* axis) principal component is applied.

**FIG 2:**
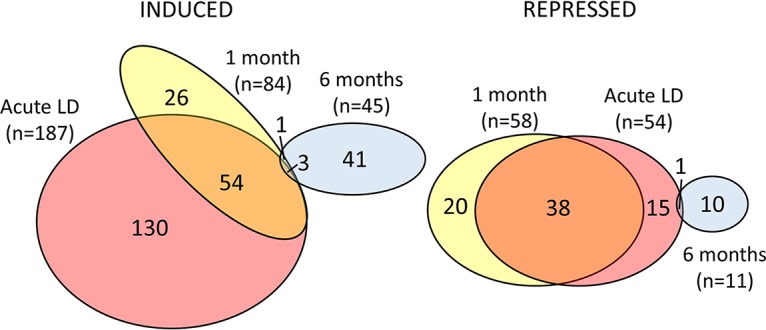
Venn diagram depicting common and unique patterns of differential gene expression among Lyme disease patients during acute LD and at 1 month or 6 months after the initiation of an appropriate antibiotic regimen. Venn diagrams were generated using a total of 335 DETs that had a fold change of at least 2, with *P* value of <0.05, relative to healthy controls. DETs for acute, 1-, and 6-month samples are represented by colored ellipses. The sizes of the ellipses are adjusted for the number of DETs in each group.

Significant differentially expressed transcripts (DETs) were defined as those having a *P* value of <0.05 and at least a 2-fold change in expression at any time point relative to the healthy donor group. A total of 335 DETs, representing 233 unique genes, were identified (see Table S1 in the supplemental material). The greatest number of DETs (241 total; 187 induced, 54 repressed) was observed in the acute phase blood samples of the LD subjects (Fig. 2). The 1-month convalescent phase samples contained 142 DETs (142 total; 84 induced, 58 repressed); most of these (92; 65%) were also differentially expressed during acute LD. Only 56 DETs (56 total; 45 induced, 11 repressed) were identified in 6-month convalescent-phase samples; of these, an overwhelming majority (51; 91%) were unique to this group. A list of the DETs with the greatest change in expression (at least 2.5-fold) is provided in Table 2, along with the corresponding fold change values for each time point.

**TABLE 2:** Top 40 genes with greatest fold changes in LD subjects relative to healthy donors

Gene symbol(s)	Gene title(s)	Entrez gene(s)	Fold change		
			Acute	1 mo	6 mo
DEFA1/DEF1B/DEF3A	Defensin, alpha 1/defensin, alpha 1B/defensin, alpha 3, neutrophil specific	1667/1668/728358	5.21	3.73	3.24
LCN2	Lipocalin 2	3934	3.95	2.59	1.00
FCGR3B	Fc fragment of IgG, low-affinity IIIb, receptor (CD16b)	2215	3.86	2.67	–1.07
MYL9	Myosin, light chain 9, regulatory	10398	3.42	2.35	–1.74
FCGR1A	Fc fragment of IgG, high-affinity Ia, receptor (CD64)	2209	3.34	1.38	1.25
CLU	Clusterin	1191	3.12	2.06	–1.76
RRM2	Ribonucleotide reductase M2	6241	3.06	1.29	–1.27
GMPR	Guanosine monophosphate reductase	2766	2.88	2.03	–1.15
IGHM	Immunoglobulin heavy constant mu	3507	2.84	2.05	–1.71
PF4	Platelet factor 4	5196	2.83	2.56	–1.27
SPARC	Secreted protein, acidic, cysteine-rich (osteonectin)	6678	2.77	2.09	–1.45
PPBP	Pro-platelet basic protein (chemokine [C-X-C motif] ligand 7)	5473	2.82	2.48	–1.22
C21orf7	Chromosome 21 open reading frame 7	56911	2.70	2.41	–1.27
TNFSF10	Tumor necrosis factor (ligand) superfamily, member 10	8743	2.77	1.87	1.43
HSPA6/HSPA7	Heat shock 70-kDa protein 6/heat shock 70-kDa protein 7	3310/3311	2.76	2.12	1.25
C6orf25	Chromosome 6 open reading frame 25	80739	2.75	2.06	–1.17
HIST1H2BK	Histone cluster 1, H2bk	85236	2.74	2.11	–1.67
MYL9	Myosin, light-chain 9, regulatory	10398	2.72	1.86	–1.45
CXCR2/CXCR2P1	Chemokine (C-X-C motif) receptor 2/chemokine (C-X-C motif) receptor 2 pseudogene 1	3579/3580	2.72	1.87	–1.12
FCGR1B	Fc fragment of IgG, high-affinity 1b, receptor (CD64)	2210	2.70	1.23	–1.06
SLC25A37	Solute carrier family 25, member 37	51312	2.68	1.88	–1.29
GBP1	Guanylate binding protein 1, interferon inducible, 67 kDa	2633	2.68	1.80	1.56
HP	Haptoglobin	3240	2.68	1.32	1.07
AIM2	Absent in melanoma 2	9447	2.67	2.19	1.42
CA2	Carbonic anhydrase II	760	2.63	2.41	–1.18
HIST1H2AG	Histone cluster 1, H2ag	8969	2.62	2.16	1.37
PTGS1	Prostaglandin-endoperoxide synthase 1 (prostaglandin G/H synthase and cyclooxygenase)	5742	2.61	2.15	–1.01
THBS1	Thrombospondin 1	7057	–4.30	–5.64	1.49
IL8	Interleukin 8	3576	–3.44	–3.88	1.79
EGR1	Early growth response 1	1958	–3.40	−2.87	1.14
G0S2	G_0_/G_1_ switch 2	50486	–3.10	–3.77	1.02
PPP1CB	Protein phosphatase 1, catalytic subunit, beta isozyme	5500	–3.02	−2.71	−1.04
NR4A2	Nuclear receptor subfamily 4, group A, member 2	4926	−2.80	–2.85	1.16
HBEGF	Heparin-binding EGF-like growth factor	1839	–2.96	–3.46	1.15
RGS1	Regulator of G-protein signaling 1	5996	–2.94	–2.70	1.12
EPPK1	Epiplakin 1	83481	–2.94	–2.44	−1.22
TNFAIP3	Tumor necrosis factor, alpha-induced protein 3	7128	–2.79	–2.51	1.08
NAMPT	Nicotinamide phosphoribosyltransferase	10135	–2.75	–3.98	1.84
CD69	CD69 molecule	969	–2.67	–2.50	−1.04
CD83	CD83 molecule	9308	−2.67	−2.50	1.29

10.1128/mBio.00047-20.1 TABLE S1 List of transcripts differentially expressed in Lyme disease patient PBMCs during acute disease and convalescence. Download Table S1, DOCX file, 0.04 MB. Copyright © 2020 Petzke et al. 2020 Petzke et al. This content is distributed under the terms of the Creative Commons Attribution 4.0 International license.

**FIG 3:**
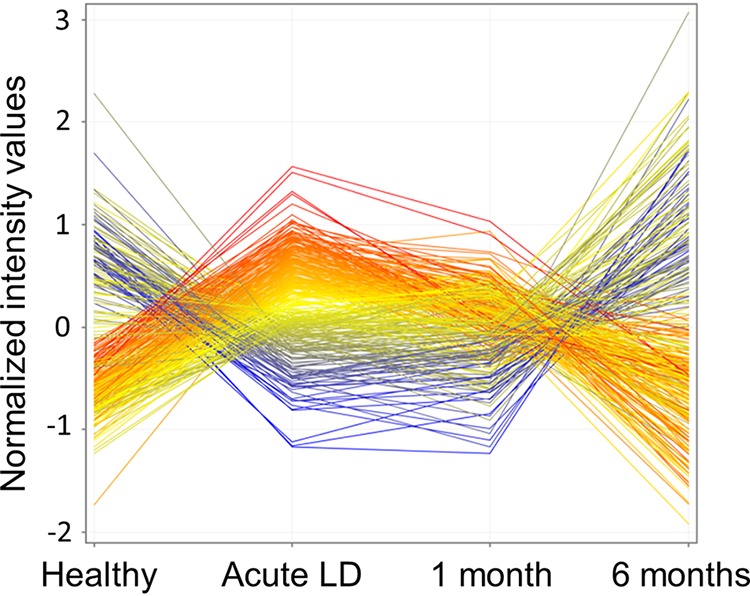
Profile plots of temporal gene expression changes in Lyme disease patients and controls. Profile plots were generated using the normalized intensities of the 335 DETs. Lines representing transcripts are colored based on the normalized expression of each transcript (blue, low; red, high) relative to the mean expression value of all transcripts in acute LD subjects.

In order to visualize temporal gene expression changes occurring during different disease states, a profile plot was generated using the normalized intensity values of the 335 DETs. Healthy donors displayed a relatively broad range in intensity values (Fig. 3); this likely reflects normal variation in gene expression in the population (27, 28). The range of normalized intensities appeared to be more restricted in the acute LD samples relative to samples from the healthy controls, likely reflecting a common response to B. burgdorferi infection among subjects. Consistent with the Venn diagrams, the profiles for acute LD and 1-month convalescent LD samples were found to be strikingly similar; however, the intensity of many of the transcripts was slightly reduced in the 1-month convalescent samples. Importantly, expression intensities for the 6-month convalescent LD samples showed greater variability in general, as was observed in healthy donors (Fig. 3). Interestingly, at 6 months convalescence, the expression levels of some transcripts that had been repressed during acute LD exceeded values observed in healthy controls. This may indicate a “rebound effect” as immune cells returned to homeostasis following clearance of the infection.

### Numerous genes involved in innate immune mechanisms are differentially expressed during acute and early convalescent LD but not during late convalescence.

**FIG 4:**
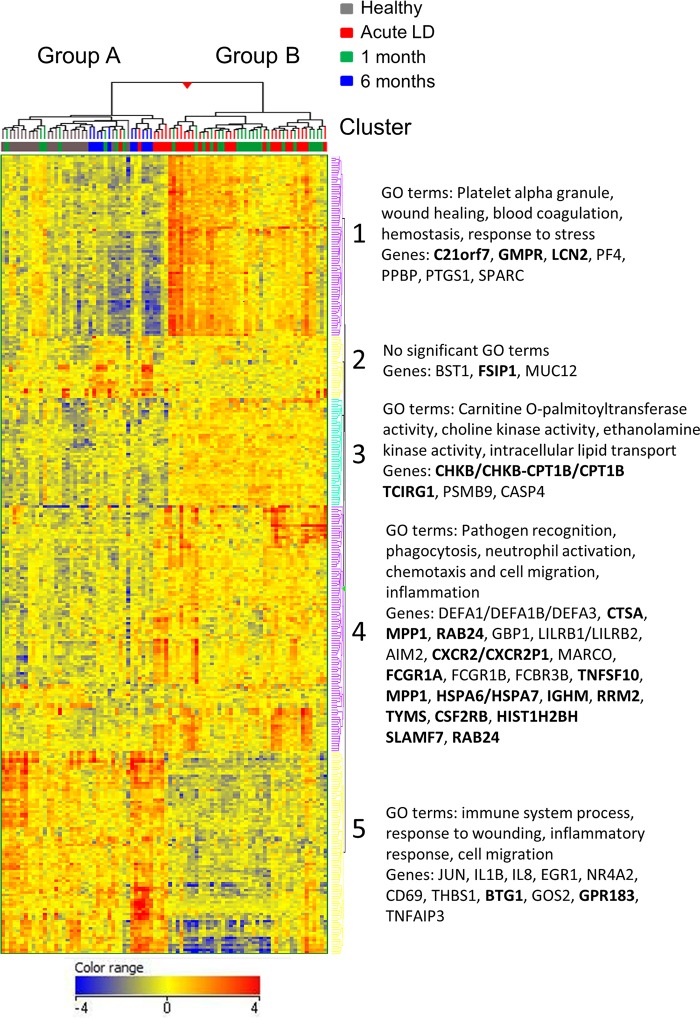
Hierarchical clustering distinguishes between disease states. Heat map with the dendrogram resulting from unsupervised hierarchical clustering performed using 335 transcripts (representing 233 genes) that were differentially expressed (at least a 2-fold change, with a *P* value of <0.05) relative to healthy controls. The values shown are normalized intensities relative to the mean. Red or blue indicates high or low expression, respectively, of the normalized intensities relative to the mean. The heat map displays five distinct clusters, three containing induced genes and two containing repressed genes. Boldfacing indicates genes that were later identified as classifiers for disease states (Tables 4 and 5). A list of the top 40 genes with greatest changes in LD subjects is presented in Table 2, and all dysregulated genes are provided in Table S1 in the supplemental material.

To further identify transcriptional patterns characteristic of disease states, the 335 DETs were used for unsupervised hierarchical clustering. As shown in Fig. 4, samples separated into two main clusters. Consistent with the principal-component analysis, all healthy donor and late-convalescent-phase samples clustered together (group A), while the majority of the acute-phase (22 of 28) and early-convalescent-phase (20 of 27) samples from LD subjects comprised a second group (group B). Four of the remaining six acute LD samples formed a small subcluster immediately adjacent to group B. One acute LD sample was distinctly separated from the other acute LD samples; this sample had been collected from the only LD subject who did not have serologic evidence of B. burgdorferi infection at any time point during the course of the study and was culture negative from skin and blood; the diagnosis of LD was based solely on the presence of MEM.

DETs separated into five gene clusters (Fig. 4 and see Table S1 in the supplemental material). Cluster 1 (54 genes, 76 probe sets) and cluster 3 (38 genes, 45 probe sets) contained genes that were strongly or moderately induced in the 42 acute and early convalescent LD samples in group B relative to healthy controls. However, increased expression of these genes was not observed in the six acute LD subjects that clustered in group A. Cluster 1 was characterized by genes involved in innate immune processes (Table S1). Significant gene ontology (GO) terms associated with cluster 1 included platelet alpha granule (*P* = 3.15E–08), wound healing (*P* = 3.28E–04), blood coagulation (*P* = 0.001), hemostasis (*P* = 0.001), and response to stress (*P* = 0.005). Cluster 3 featured genes involved in fatty acid catabolism (Table S1). Significant GO terms included carnitine *O*-palmitoyltransferase activity (*P* = 2.72E–04), choline kinase activity (*P* = 2.72E–04), ethanolamine kinase activity (*P* = 2.72E–04), and intracellular lipid transport (*P* = 5.32E–04).

The majority of acute LD subjects showed a significant induction of genes in cluster 4. This result contrasted with that for clusters 1 and 3, where different responses were observed for the acute LD subjects in group A and group B. Of the 69 transcripts in cluster 4, 28 (41%) are involved in innate immune cell functions, including pathogen recognition, phagocytosis, neutrophil activation, chemotaxis and cell migration, and inflammation. The most highly induced transcript encodes DEFA1/DEFA1B/DEFA3 (defensin, alpha 1/defensin, alpha 1B/defensin, alpha 3, neutrophil specific), microbicidal proteins of neutrophil granules that effectively kill B. burgdorferi *in vitro* (29) (Table 2). With the single exception of DEFA1/DEFA1B/DEFA3, which was upregulated at all time points, genes in cluster 4 were significantly induced only during acute and early convalescent LD and returned to levels observed in the healthy donors within 6 months (Table 2).

Cluster 2 contained 22 genes (26 probe sets) that, with three exceptions, were not significantly changed during acute or early convalescent LD but were significantly induced in the late convalescent LD (6 months) subjects. Cluster 5 consisted of transcripts for 50 genes that were significantly repressed in the majority of acute and early convalescent LD patients relative to healthy subjects. Significant GO terms for these genes included immune system process (*P* = 4.98E–06), response to wounding (*P* = 1.21E–05), and cell migration (*P* = 2.92E–04).

### Interferon-regulated genes characterize the response to acute disseminated *B. burgdorferi* infection.

Interferome (http://www.interferome.org/interferome/home.jspx), a database of interferon (IFN)-regulated genes (30), was employed to analyze the genes dysregulated during acute LD. The following parameters were applied to the analysis: human (species), hematopoietic/immune (system), and blood (organ). Totals of 106 of 131 (81%) induced genes (encoded by 187 transcripts) and 25 of 30 (83%) of the repressed genes (encoded by 54 transcripts) were identified as interferon regulated. These included 32 of the 40 genes with the greatest expression changes (Table 2).

### Normalization of transcriptome following treatment is concordant with resolution of symptoms.

LD subjects were questioned regarding symptoms at each visit. Symptoms that had existed due to a preexisting condition were not included in the analysis. At the initial visit, 82% of subjects with acute LD reported experiencing at least one symptom (Table 3). Fatigue was the most commonly reported symptom (68%), followed by headache (47%), arthralgia (42%), myalgia (40%), and stiff neck (34%). Strikingly, only approximately one-half as many subjects (43%) reported experiencing any symptoms at the second visit. Fatigue remained the most commonly reported symptom (23%), followed by arthralgia (11%), myalgia (11%), and stiff neck. Only 3% of subjects at the second visit reported headache. Of 11 evaluable subjects at 6 months after antibiotic treatment, only 1 (9%) reported experiencing any symptoms (arthralgia).

**TABLE 3:** Reported symptoms of LD subjects before and after antibiotic therapy

Symptom	No./total no. (%)		
	Acute LD	Convalescent LD	
		1 mo	6 mo
Arthralgia	16/38 (42)	4/35 (11)	1/11 (9)
Dizziness	7/38 (18)	1/35 (3)	0/11 (0)
Fatigue	26/38 (68)	8/35 (23)	0/11 (0)
Headache	18/38 (47)	1/35 (3)	0/11 (0)
Myalgia	15/38 (40)	4/35 (11)	0/11 (0)
Stiff neck	13/38 (34)	4/35 (11)	0/11 (0)
Any symptom present	31/38 (82)	15/35 (43)	1/11 (9)

### Identification and validation of predictor genes.

**FIG 5:**
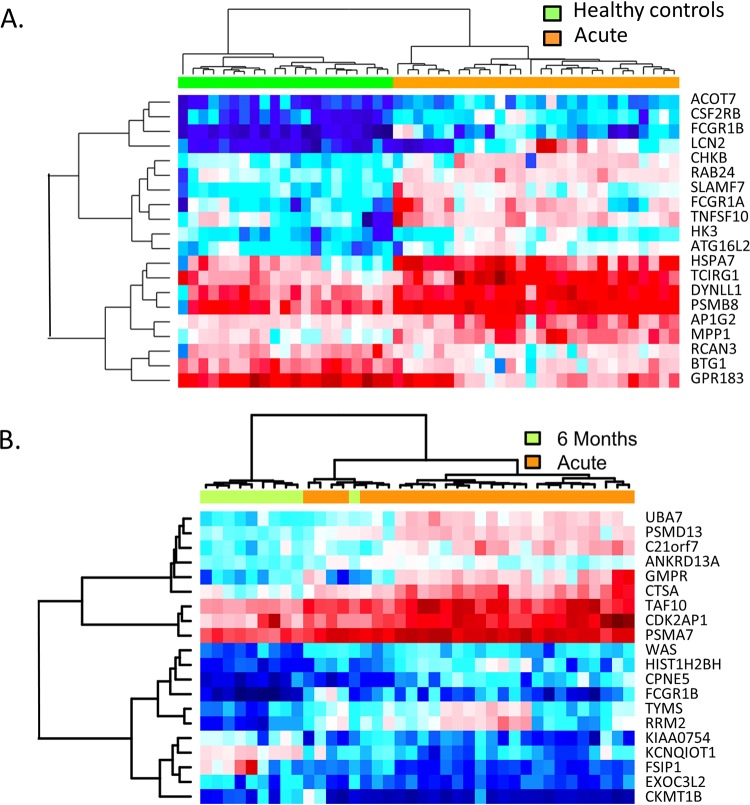
Twenty-gene classifier sets identified by random forest analysis accurately distinguish between disease states. (A) Hierarchical clustering was performed with samples from acute LD subjects (orange) and healthy donors (green) based on normalized expression intensities of 20 genes having the highest random forest importance levels for these groups (shown on right and in Table 4). (B) A second unique set of 20 genes (shown on the right and in Table 5) having the highest random forest importance levels when comparing acute LD subjects (orange) and 6-month convalescent LD subjects (green) was used for hierarchical clustering of samples from these groups.

**FIG 6:**
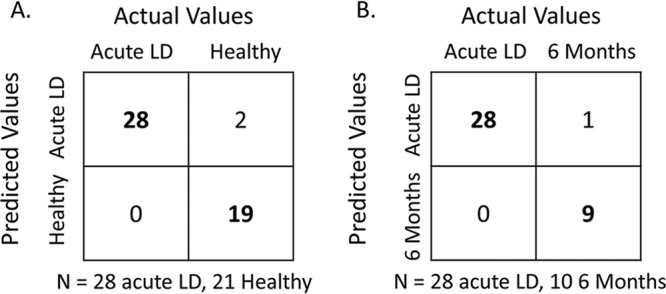
Performance of 20-gene classifier sets identified by random forest analysis. Separate leave-one-out cross-validation experiments were performed using the distinct 20-gene classifier sets shown in Tables 4 and 5, respectively, for comparison of subjects with acute LD to (A) healthy controls and (B) 6-month convalescent LD subjects. The results are presented as confusion matrices with boldfacing indicating the samples that were correctly classified.

One major limitation of serological tests is the inability to detect infection prior to the appearance of antibodies. A predictive model was developed based on application of the random forest algorithm to the 2004 most highly variable genes in three data sets (acute LD, 6-month convalescent LD, and healthy controls). In the first comparison, the capability of this model to correctly distinguish between subjects with acute LD and healthy controls was determined and the top 20 genes with the highest random forest importance levels were identified (Table 4). Hierarchical clustering using only these 20 genes accurately separated acute LD subjects and healthy controls into two distinct clusters (Fig. 5A). Moreover, this 20-gene classifier set correctly distinguished subjects with acute LD from healthy donors with 100% sensitivity and 96% accuracy (correct predictions/test set size) (Fig. 6). In comparison, only 22/27 of these subjects tested positive by ELISA for B. burgdorferi-specific antibodies at the initial visit, resulting in 81% sensitivity for the serology-based test. Four of the five patients who were seronegative by ELISA at the initial visit seroconverted by the time of the second visit.

**TABLE 4:** Top 20 classifier genes that discriminate subjects with acute LD from healthy controls

Gene symbol	Gene title	RFIL (%)^*a*^
PSMB8	Protease subunit β8	9.14
SLAMF7	SLAM family member 7	7.58
RAB24	RAB24, member RAS oncogene family	7.11
FCGR1B	Fc fragment of IgG, high affinity 1b, receptor (CD64)	6.52
MPP1	Membrane protein, palmitoylated 1, 55 kDa	5.86
CSF2RB	Colony stimulating factor 2 receptor, beta, low affinity (granulocyte-macrophage)	5.55
TNFSF10	Tumor necrosis factor (ligand) superfamily, member 10	4.75
BTG1	B-cell translocation gene 1, antiproliferative	4.72
GPR183	G protein-coupled receptor 183	4.54
ATG16L2	Autophagy-related 16-like 2	4.50
ACOT7	Acyl-CoA thioesterase 7	4.37
TCIRG1	T-cell, immune regulator 1, ATPase, H^+^ transporting V0 subunit a3	4.25
CHKB_CPT1B	CHKB-CPT1B readthrough (NMD candidate)	4.20
DYNLL1	Dynein light chain LC8-type 1	4.13
LCN2	Lipocalin 2	4.05
HSPA6_HSP70B′	Heat shock protein family A (Hsp70) member 6	4.02
FCGR1A	Fc fragment of IgG, high-affinity 1a, receptor (CD64)	3.85
RCAN3	RCAN family member 3 (calcipressin 3)	3.74
HK3	Hexokinase 3	3.65
AP1G2	Adaptor-related protein complex 1 γ2 subunit	3.48
Total		100

a RFIL, random forest importance level.

Another major limitation of most serologic diagnostic tests is the inability to distinguish between active and prior infection as circulating antibodies are present long after the pathogen is cleared. Application of the random forest algorithm to samples from LD subjects at baseline and at 6-month convalescence resulted in a separate distinct set of 20 classifier genes (Table 5). Hierarchical clustering of samples using this unique 20-gene classifier set correctly categorized the preponderance of samples from these two groups (Fig. 5B). In addition, acute LD could be discriminated from 6-month convalescent subjects with 100% sensitivity and 97% accuracy (Fig. 6).

**TABLE 5:** Top 20 classifier genes that distinguish between acute and 6-month convalescent LD subjects

Gene symbol	Gene name	RFIL (%)^*a*^
TAF10	TATA-box binding protein associated factor 10	9.96
CTSA	Cathepsin A	9.26
EXOC3L2	Exocyst complex component 3-like 2	6.77
RRM2	Ribonuclease reductase regulatory subunit M2	5.99
PSMA7	Proteasome subunit alpha 7	5.91
KCNQ1OT1	KCNQ1 opposite strand/antisense transcript 1 (nonprotein coding)	5.55
CKMT1B	Creatine kinase, mitochondrial 1B	5.34
ANKRD13A	Ankyrin repeat domain 13A	4.86
UBA7	Ubiquitin-like modifier activating enzyme 7	4.71
CDK2AP1	Cyclin-dependent kinase 2 associated protein 1	4.53
TYMS	Thymidylate synthetase	4.51
FSIP1	Fibrous sheath interacting protein 1	3.92
KIAA0754	Microtubule-actin crosslinking factor 1	3.79
HIST1H2BH	Histone cluster 1 H2B family member H	3.76
FCGR1B	Fc fragment of IgG, high-affinity Ib, receptor (CD64)	3.73
WAS	Wiskott-Aldrich syndrome gene	3.71
CPNE5	Copine 5	3.48
C21orf7	Chromosome 21 open reading frame 7	3.46
GMPR	Guanosine monophosphate reductase	3.38
PSMD13	Proteasome 26S subunit, non-ATPase 13	3.36
Total		100

a RFIL, random forest importance level.

Validation of the specificity of the classifier gene set was performed by applying the prediction model to a published microarray data set generated using peripheral blood mononuclear cells (PBMCs) from patients with acute infections caused by common bacterial and viral pathogens: Staphylococcus aureus, Streptococcus pneumoniae, Escherichia coli, or influenza A virus (17). First, the top 10% of genes with the greatest variance were selected. Next, iterations (*n* = 10) of the random forest algorithm were run to identify the top 20 genes associated with each infectious agent that had the highest importance levels (Table 6). Using these 20-gene classifier sets, random forest analysis correctly identified patients with specific infections with prediction accuracies of 100% (influenza A virus), 98% (B. burgdorferi), 95% (S. pneumoniae and S. aureus), and 94% (E. coli). Comparison of the 20-gene sets revealed that acute infections due to E. coli, S. aureus, and S. pneumoniae shared multiple classifiers; the greatest number (eight) of shared classifiers was between E. coli and S. aureus infections (Table 6). The gene lists were analyzed for IFN-responsive genes using Interferome as described above. The only classifier sets that contained more than one IFN-regulated gene were those for B. burgdorferi (*n* = 15) and influenza A (*n* = 6) (Table 6). Remarkably, however, all 20 classifier genes for acute infection with B. burgdorferi were unique to that organism; none was shared with any of the other bacterial infections or with infection due to influenza A.

**TABLE 6:** Twenty-gene classifier sets distinguish B. burgdorferi infection from acute infections caused by other bacterial and viral pathogens^*a*^

E. coli	S. aureus	S. pneumoniae	B. burgdorferi (acute LD)	Influenza A virus
**ELANE**	**ELANE**	**SERPINB2**	PSMB8*	IFI27*
**CEACAM8**	**DEFA1/DEFA1B/DEFA3**	RNASE3	SLAMF7*	SIGLEC1*
**IL8**	C21orf59	DEFA4	RAB24*	OTOF
**MMP8**	**MGAM**	CHIT1	FCGR1B*	RSAD2*
**OLFM4**	ADM*	**ELANE**	MPP1*	CD1C
**DEFA1/DEFA1B/DEFA3**	**LTF**	AZU1	CSF2RB*	IFI44L*
**MGAM**	**MPO**	**CXCL2**	TNFSF10*	RPS4Y1
FOSB	BPI	RNASE2*	BTG1*	AKR7A2
**AHSP**	SCN3A	FCGBP	GPR183*	IFIT3*
HBG1/HBG2/	CCDC99	**CEACAM8**	ATG16L2*	CACNA2D3
SELENBP1	**AHSP**	CAMP	ACOT7*	LAMP3*
AKR1C3	DUSP3	**ANXA3**	TCIRG1	EPHB2
**CXCL2**	**MMP8**	**DEFA1/DEFA1B/DEFA3**	CHKB_CPT1B	MCM10
ALAS2	**CEACAM8**	PGLYRP1	DYNLL1	ABHD8
LMAN2L	CD14	**IL8**	LCN2	KIF23
**LTF**	**OLFM4**	CEACAM6	HSPA6_HSP70B′	HLA-DQA1/LOC100507718/LOC100509457
RRP1	NPL	EPHA4	FCGR1A*	MX2
CCL27	MARCO	COL9A3	RCAN3*	BTF3P11
HBD	**ANXA3**	CHI3L1	HK3*	AKR1B10
ZNF639	PLBD1	**MPO**	AP1G2*	PLK1S1

a Genes are listed in order of random forest analysis importance level (highest to lowest). *, interferon-regulated gene. Genes that appear on the classifier list for more than one infectious agent are designated in boldface.

## DISCUSSION

In this study, multiple approaches were used to identify a peripheral blood signature that would enable reliable detection of early disseminated LD at a time point when standard serologic testing may be suboptimally sensitive. A 20-gene classifier set that correctly distinguished subjects with acute LD from healthy donors with 96% accuracy, 100% sensitivity, and 90% specificity was identified. A second major limitation of antibody-based tests is the inability to differentiate between acute infection and resolved infection (after antibiotic treatment) due to specific circulating antibodies that may persist for years after the microbe has been eliminated. The identified 20-gene classifier set was able to discriminate acute LD from 6-month convalescent subjects with 97% accuracy, 100% sensitivity, and 90% specificity. Notably, gene expression changes corresponded to reported symptoms. The greatest number of genes with altered expression was present in the acute LD group; symptoms were reported by 82% of all acute LD subjects in this study and by 93% of the 28 subjects whose blood was analyzed for gene expression. In contrast, return of the gene expression profile to that observed in the healthy donors corresponded with resolution of symptoms: only one 6-month LD convalescent subject (9%) reported having any symptom. Thus, the identified classifier set has the potential for serving as a test for disease resolution.

The algorithm used to generate the classifier gene set for acute B. burgdorferi infection was applied to published microarray data sets for PBMCs collected from patients with acute infections caused by three common bacterial pathogens or by influenza A virus. Importantly, all 20 classifier genes for acute B. burgdorferi infection were completely unique and were not associated with any of these four pathogens. Therefore, the gene classifier sets described here not only demonstrated high sensitivity for acute LD relative to healthy donors and convalescent LD patients, but the 20-gene classifier set for acute LD distinguished B. burgdorferi infection from the other tested bacterial or viral infections with 100% specificity.

In sharp contrast to the gene classifiers for the other three bacterial pathogens, the classifier gene sets for B. burgdorferi and influenza A infection were both characterized by an IFN-regulated signature, although the individual genes comprising each set were unique. *IFI27* (interferon alpha inducible protein 27) is the classifier gene for influenza A that has the highest random forest importance value. *IFI27* has been described in a separate study as a novel single-gene biomarker in patient blood that was able to discriminate, with 88% diagnostic accuracy and 90% specificity, between influenza virus- and bacterium-associated respiratory infections (31). We have previously demonstrated that B. burgdorferi induces numerous IFN-regulated genes in skin at the site of an EM lesion (32), many of which were also dysregulated in Lyme disease patient PBMCs in the present study. Of note, the 20-gene classifier set for B. burgdorferi infection included 15 IFN-regulated genes; five were also significantly induced in EM skin biopsy specimens from patients with disseminated Lyme disease (32). Several of these genes encode proteins involved in pathogen recognition and phagocytosis, and antigen processing, including: the Fc gamma receptors FCGR1A and FCGR1B (Fc fragment of IgG, high-affinity 1a and 1b, receptor [CD64]), TNFSF10 (tumor necrosis factor [ligand] superfamily, member 10), and PSMB8 (proteasome subunit beta 8). Interestingly, the classifier gene sets for infections caused by each of the other three bacterial pathogens evaluated were nearly devoid of IFN-regulated genes, with none associated with E. coli infection and one IFN-regulated gene each associated with S. aureus and S. pneumoniae infections. Collectively, these results confirm and extend our previous observation that B. burgdorferi elicits an IFN-dominated transcriptional signature during early infection, a sharp distinction from the immunological footprints generated by the other bacterial pathogens examined. In addition, the 20-gene classifier set clearly distinguishes B. burgdorferi infection from that caused by influenza A, although both pathogens potently stimulate the interferon signaling pathway (33).

Bouquet and colleagues also examined the transcriptional profile in PBMCs of LD patients with EM before antibiotic treatment, 3 weeks later, and then 6 months after the completion of antibiotic therapy (34). There is general consensus between Bouquet et al. and a major finding of the present study: acute infection with B. burgdorferi elicits a distinct gene expression profile in patient blood that persists for at least 3 weeks after infection. However, in contrast to Bouquet et al., we observed that the majority of differentially regulated genes return to healthy donor levels by 6 months posttreatment. There are several differences between the two studies that might explain the discrepancies in the findings. The most significant difference may be in the patient population under investigation. The present study was restricted to subjects with definitive early disseminated LD. A total of 95% of enrolled LD subjects had either MEM (67%) and/or positive blood culture for B. burgdorferi (74%); the remaining subject had facial palsy, a sign of disseminated LD. The inclusion criteria of Bouquet et al. were less stringent and consisted of a physician-documented EM of >5 cm with at least one concurrent nonspecific symptom (headache, fever, chills, fatigue, and/or new muscle or joint pains). Cultivation of B. burgdorferi from any clinical samples was not reported, and only 43% of LD subjects had MEM. Of the 29 subjects with LD in the Bouquet et al. study, 8 did not seroconvert, and 1 was not tested. In addition to the enrollment criteria, the definition for altered gene expression differed between the studies; Bouquet et al. used a 1.5-fold change cutoff compared to the 2-fold change in the present study. Significantly, in the present study, random forest analysis was employed to build predictive models. Classifier gene sets that could separately distinguish healthy controls from patients with acute disseminated infection, and between such patients and those with resolved infection, were identified.

It is important to note the limitations of the current investigation. It was not completely longitudinal and included a relatively small sample size for the 6-month visit. This was primarily due to the fact that 6-month samples were not collected during the first 2 years of the study; the 6-month convalescent time point was added when it became apparent that transcript levels had not returned to normal by 1 month posttreatment. In addition, some study subjects were lost to follow-up, and some RNA samples did not meet the quality requirements for microarray hybridization. A sample size of 10, however, has proven to be sufficient for rigorous statistical comparison with earlier time points and with healthy donors in other studies (18). Since only one of the 10 subjects reported having any symptoms at 6 months, the small sample pool was insufficient for identifying potential transcriptome alterations associated with persisting symptoms. Another limitation is the specific focus on patients with definitive evidence of disseminated infection. An optimal diagnostic test for LD should be able to detect infection at its earliest stages, when B. burgdorferi is still localized to the skin. Current studies are under way to test the sensitivity of the diagnostic biomarker set using samples from subjects with EM, but without evidence of dissemination. It should also be noted, that the use of published data sets rather than prospectively collected samples (as in Table 6) could potentially lead to artifacts in the cross-comparisons.

In conclusion, we report the development, using gene expression data, of an efficient computational framework to generate a 20-gene classifier set that detects disseminated B. burgdorferi infection with high sensitivity and specificity. This unique classifier set may have a critical advantage over current serologic tests in that it accurately discriminated between active and resolved infection. This computational approach offers the potential for more accurate diagnosis of early disseminated Lyme disease. It may also allow improved monitoring of treatment efficacy and disease resolution.

## MATERIALS AND METHODS

### Study subjects.

All subjects were adult volunteers of at least 18 years of age and provided written informed consent prior to sample collection, in accordance with the study protocol approved by the Institutional Review Board of New York Medical College (NYMC). Healthy donors were recruited from NYMC staff, excluding members of the investigators’ laboratories, and met the following inclusion criteria: no history of LD, no receipt of a Lyme disease vaccine, no evidence of a current infectious disease, not pregnant, and no usage of an immunosuppressive medication. Patients were recruited from the Lyme Disease Diagnostic Center of NYMC during the summer seasons of 2005 to 2006 and 2010 to 2013. Blood samples were collected at the time of diagnosis (acute LD) and at approximately 1 and 6 months after the initiation of a recommended course of antibiotics (7). Serologic testing of LD subjects for antibodies to B. burgdorferi was performed by a whole-cell sonicate ELISA. Serologic testing of healthy controls for antibodies to B. burgdorferi was performed once by IgG immunoblot. Analysis was restricted to samples collected from individuals with objective evidence of dissemination, most often based on the presence of multiple erythema migrans (MEM) skin lesions and/or the cultivation of B. burgdorferi from blood, as previously described (35).

### Blood collection and RNA isolation.

Venous blood was collected directly into BD-Vacutainer CPT tubes (Becton Dickinson, Franklin Lakes, NJ). PBMCs were isolated by centrifugation, according to the manufacturer’s protocol, no later than 3 h after blood collection. PBMCs were washed with Hanks’ balanced salt solution without calcium, magnesium, or phenol red (Gibco-BRL, Grand Island, NY), and RNA was isolated immediately thereafter under RNase-free conditions using the PureScript total RNA isolation kit (Gentra, Minneapolis, MN) or the Ambion ToTALLY RNA isolation kit (Life Technologies, Grand Island, NY), according to the manufacturers’ instructions. Contaminating DNA was removed using the DNA-free kit (Ambion, Austin, TX). RNA was eluted in 20 μl RNase/DNase-free water and stored at –80°C after the addition of 32 U of RNase inhibitor (Promega, Madison, WI). RNA integrity was assessed by electrophoresis using an Agilent Bioanalyzer 2100 (Agilent, Palo Alto, CA) prior to cDNA synthesis for microarray hybridization. Samples having an RNA integrity number below 6 were excluded from further analysis.

### Microarray hybridization.

Between 5 and 20 ng of total RNA from each PBMC sample was used to generate high-fidelity cDNA using an Ovation RNA amplification system (NuGEN Technologies, Inc., San Carlos, CA) according to the manufacturer’s protocol. The amplified cDNA was fragmented to 50 to 100 nucleotides, labeled with biotin, and hybridized to the Affymetrix GeneChip.HG-U219 high-density oligonucleotide array (Affymetrix, Santa Clara, CA). After hybridization, the arrays were stained with streptavidin-phycoerythrin and washed in an Affymetrix fluidics module using standard Affymetrix protocols. The detection and quantitation of target hybridization was performed using a GeneArray Scanner 3000 (Affymetrix). All procedures were performed at the Bionomics Research and Technology Center, Rutgers University, Piscataway, NJ.

### Microarray data analysis.

Microarray data were analyzed using GeneSpring GX14.9 software (Agilent Technologies, Santa Clara, CA). Raw expression values in CEL file format were normalized by robust multiarray analysis (RMA) and quantile normalization, filtered to include only those with intensity values above the 20th percentile, and baseline transformed to the median of all samples. Statistical analysis was performed using one-way analysis of variance with Benjamini-Hochberg multiple testing correction to reduce false positives (36). Differentially expressed transcripts, defined as those having a *P* value of <0.05 and a fold change of at least 2 relative to the healthy donor group, were subjected to hierarchical clustering and principal-component analysis.

### Predictive modeling.

A generic predictive modeling framework was developed and applied to two comparisons: acute LD (*n* = 28) versus healthy donors (*n* = 21) and acute LD versus 6-month convalescent LD (*n* = 10). In the first step, the distribution of the gene expression variance across all experimental groups was computed, and genes with variance at or above the 90th percentile were identified. This threshold is a parameter of the framework and can be appropriately set based on the variance distribution in a considered cohort of samples. In the second step, expression data containing the top 10% of variance in each experimental group were subjected to iterations (*n* = 50) of random forest analysis, a well-established machine learning algorithm (37). An importance value for each gene was generated following each iteration of random forest analysis, and a final importance value for each gene was computed by averaging the importance values across all 50 iterations. Averaged importance values were used to rank all top selected genes. Finally, for each experiment, leave-one-out predictive modeling was performed, as well as tested using incrementally expanding sets of the most significant genes (top 20 through top 2004), to assess the changes in accuracy performance across different sets of predictors.

### Comparison of classifier genes for LD and other infectious diseases.

Microarray-based transcriptome data set GSE6269, containing gene expression profiles from PBMCs from patients with acute infections due to Escherichia coli, Staphylococcus aureus, Streptococcus pneumoniae, or influenza A virus (17) was downloaded from the GEO database and subjected to random forest analysis using the same framework and parameters that were applied to the LD data.

### Data availability.

The transcriptome data obtained in this study have been submitted to the Gene Expression Omnibus (GEO) data repository under accession number GSE145974.
