# Supplementary material for: Global Transcriptome Analysis Identifies a Diagnostic Signature for Early Disseminated Lyme Disease and Its Resolution
Source: mBio. 2020 Mar 17;11(2):e00047-20. doi: 10.1128/mBio.00047-20 (PMC7078463; doi:10.1128/mBio.00047-20)
Supplement: TABLE S1 [file mBio.00047-20-st001.docx]

**Supplementary Table 1.** List of transcripts differentially expressed in Lyme disease patient PBMCs during disease and convalescence

| **Cluster** | **Probe Set ID** | **Entrez Gene** | **Acute LD^a^** | **1 month conv^a^** | **6 months conv^a^** | **Gene Symbol** | **Gene Title** |
| --- | --- | --- | --- | --- | --- | --- | --- |
| **1** | 11715268_s_at | 2812 /// 5413 | 2.17 | 1.79 | -1.40 | GP1BB /// SEPT5 | glycoprotein Ib (platelet), beta polypeptide /// septin 5 |
| **1** | 11715413_s_at | 8848 | 2.04 | 1.72 | -1.32 | TSC22D1 | TSC22 domain family, member 1 |
| **1** | 11715588_x_at | 10398 | 3.42 | 2.35 | -1.74 | MYL9 | myosin, light chain 9, regulatory |
| **1** | 11715603_s_at | 6678 | 2.89 | 2.35 | -1.70 | SPARC | secreted protein, acidic, cysteine-rich (osteonectin) |
| **1** | 11715604_x_at | 6678 | 2.80 | 2.20 | -1.57 | SPARC | secreted protein, acidic, cysteine-rich (osteonectin) |
| **1** | 11717090_a_at | 8349 | 2.18 | 1.44 | -1.05 | HIST2H2BE | histone cluster 2, H2be |
| **1** | 11717099_at | 85236 | 2.74 | 2.11 | -1.67 | HIST1H2BK | histone cluster 1, H2bk |
| **1** | 11717193_a_at | 100128731 | 2.20 | 2.00 | -1.10 | OST4 | oligosaccharyltransferase 4 homolog (S. cerevisiae) |
| **1** | 11718446_at | 83699 | 2.49 | 2.15 | -1.70 | SH3BGRL2 | SH3 domain binding glutamic acid-rich protein like 2 |
| **1** | 11718447_s_at | 83699 | 2.04 | 1.83 | -1.83 | SH3BGRL2 | SH3 domain binding glutamic acid-rich protein like 2 |
| **1** | 11718445_at | 83699 | 2.02 | 1.77 | -1.71 | SH3BGRL2 | SH3 domain binding glutamic acid-rich protein like 2 |
| **1** | 11718448_at | 83699 | 2.01 | 1.55 | -1.47 | SH3BGRL2 | SH3 domain binding glutamic acid-rich protein like 2 |
| **1** | 11718604_a_at | 222166 | 2.06 | 2.08 | -1.28 | C7orf41 | chromosome 7 open reading frame 41 |
| **1** | 11718787_at | 4900 | 2.29 | 1.92 | -1.27 | NRGN | neurogranin (protein kinase C substrate, RC3) |
| **1** | 11720202_at | 2766 | 2.88 | 2.03 | -1.15 | GMPR | guanosine monophosphate reductase |
| **1** | 11721184_at | 340348 | 1.90 | 2.12 | -1.59 | TSPAN33 | tetraspanin 33 |
| **1** | 11721837_a_at | 4638 | 2.01 | 1.64 | -1.35 | MYLK | myosin light chain kinase |
| **1** | 11722209_a_at | 5742 | 2.61 | 2.15 | -1.01 | PTGS1 | prostaglandin-endoperoxide synthase 1 (prostaglandin G/H synthase and cyclooxygenase) |
| **1** | 11722208_a_at | 5742 | 2.24 | 1.98 | -1.15 | PTGS1 | prostaglandin-endoperoxide synthase 1 (prostaglandin G/H synthase and cyclooxygenase) |
| **1** | 11722379_at | 2791 | 2.40 | 2.32 | -1.57 | GNG11 | guanine nucleotide binding protein (G protein), gamma 11 |
| **1** | 11724021_at | 3017 | 2.04 | 1.57 | -1.30 | HIST1H2BD | histone cluster 1, H2bd |
| **1** | 11725428_a_at | 22885 | 2.05 | 1.55 | -1.29 | ABLIM3 | actin binding LIM protein family, member 3 |
| **1** | 11725694_at | 8436 | 2.02 | 1.77 | -2.22 | SDPR | serum deprivation response |
| **1** | 11727602_x_at | 5196 | 2.83 | 2.56 | -1.27 | PF4 | platelet factor 4 |
| **1** | 11728137_at | 7504 | 2.08 | 1.45 | -1.17 | XK | X-linked Kx blood group (McLeod syndrome) |
| **1** | 11728717_at | 6374 | 2.14 | 2.02 | -1.18 | CXCL5 | chemokine (C-X-C motif) ligand 5 |
| **1** | 11728719_a_at | 4052 | 2.04 | 1.76 | -1.70 | LTBP1 | latent transforming growth factor beta binding protein 1 |
| **1** | 11729063_a_at | 5154 | 2.04 | 1.96 | -1.40 | PDGFA | platelet-derived growth factor alpha polypeptide |
| **1** | 11729759_at | 64407 | 2.34 | 2.00 | -1.03 | RGS18 | regulator of G-protein signaling 18 |
| **1** | 11729758_at | 64407 | 2.03 | 1.69 | -1.05 | RGS18 | regulator of G-protein signaling 18 |
| **1** | 11730260_a_at | 5577 | 2.28 | 1.77 | -1.10 | PRKAR2B | protein kinase, cAMP-dependent, regulatory, type II, beta |
| **1** | 11730261_a_at | 5577 | 2.03 | 1.63 | -1.79 | PRKAR2B | protein kinase, cAMP-dependent, regulatory, type II, beta |
| **1** | 11731161_at | 3674 | 2.27 | 1.59 | -1.23 | ITGA2B | integrin, alpha 2b (platelet glycoprotein IIb of IIb/IIIa complex, antigen CD41) |
| **1** | 11731160_a_at | 3674 | 2.12 | 1.38 | -1.26 | ITGA2B | integrin, alpha 2b (platelet glycoprotein IIb of IIb/IIIa complex, antigen CD41) |
| **1** | 11731693_at | 3012 | 2.09 | 2.14 | -1.10 | HIST1H2AE | histone cluster 1, H2ae |
| **1** | 11731996_at | 25893 | 2.29 | 1.60 | -1.33 | TRIM58 | tripartite motif-containing 58 |
| **1** | 11732453_s_at | 3081 | 2.15 | 2.31 | 1.09 | HGD | homogentisate 1,2-dioxygenase |
| **1** | 11732552_a_at | 219670 | 2.55 | 2.94 | -1.50 | ENKUR | enkurin, TRPC channel interacting protein |
| **1** | 11733115_a_at | 760 | 2.63 | 2.41 | -1.18 | CA2 | carbonic anhydrase II |
| **1** | 11735066_at | 3690 | 2.03 | 1.56 | -1.41 | ITGB3 | integrin, beta 3 (platelet glycoprotein IIIa, antigen CD61) |
| **1** | 11738047_a_at | 79993 | 2.21 | 1.67 | -1.17 | ELOVL7 | ELOVL family member 7, elongation of long chain fatty acids (yeast) |
| **1** | 11738048_a_at | 79993 | 2.14 | 1.71 | -1.01 | ELOVL7 | ELOVL family member 7, elongation of long chain fatty acids (yeast) |
| **1** | 11739745_a_at | 27030 | 2.51 | 2.78 | -1.53 | MLH3 | mutL homolog 3 (E. coli) |
| **1** | 11740538_at | 147199 /// 653486 | 2.39 | 2.09 | 1.31 | LOC653486 /// SCGB1C1 | secretoglobin, family 1C, member 1-like /// secretoglobin, family 1C, member 1 |
| **1** | 11741884_a_at | 54498 | 2.04 | 1.59 | -1.35 | SMOX | spermine oxidase |
| **1** | 11743087_at | 5473 | 2.82 | 2.48 | -1.22 | PPBP | pro-platelet basic protein (chemokine (C-X-C motif) ligand 7) |
| **1** | 11745803_x_at | 7168 | 2.33 | 1.79 | -1.14 | TPM1 | tropomyosin 1 (alpha) |
| **1** | 11746124_x_at | 56911 | 3.08 | 2.63 | -1.29 | C21orf7 | chromosome 21 open reading frame 7 |
|  |  |  |  |  |  |  |  |
| **1** | 11746554_a_at | 6678 | 2.76 | 2.26 | -1.78 | SPARC | secreted protein, acidic, cysteine-rich (osteonectin) |
| **1** | 11746555_x_at | 6678 | 2.48 | 1.96 | -1.62 | SPARC | secreted protein, acidic, cysteine-rich (osteonectin) |
| **1** | 11746560_a_at | 340205 | 2.29 | 1.70 | -1.15 | TREML1 | triggering receptor expressed on myeloid cells-like 1 |
| **1** | 11746740_a_at | 2017 | 2.09 | 1.85 | -1.32 | CTTN | cortactin |
| **1** | 11747047_a_at | 56911 | 2.50 | 2.29 | -1.25 | C21orf7 | chromosome 21 open reading frame 7 |
| **1** | 11748570_a_at | 10826 | 2.02 | 1.64 | -1.65 | C5orf4 | chromosome 5 open reading frame 4 |
| **1** | 11749730_a_at | 5742 | 2.05 | 1.54 | -1.23 | PTGS1 | prostaglandin-endoperoxide synthase 1 (prostaglandin G/H synthase and cyclooxygenase) |
| **1** | 11749774_x_at | 56911 | 2.54 | 2.37 | -1.27 | C21orf7 | chromosome 21 open reading frame 7 |
| **1** | 11751644_a_at | 57326 | 2.06 | 1.92 | -1.03 | PBXIP1 | pre-B-cell leukemia homeobox interacting protein 1 |
| **1** | 11752423_a_at | 2162 | 2.18 | 1.54 | -1.15 | F13A1 | coagulation factor XIII, A1 polypeptide |
| **1** | 11754059_x_at | 6678 | 2.74 | 2.05 | -1.51 | SPARC | secreted protein, acidic, cysteine-rich (osteonectin) |
| **1** | 11754058_a_at | 6678 | 2.58 | 2.01 | -1.33 | SPARC | secreted protein, acidic, cysteine-rich (osteonectin) |
| **1** | 11754084_x_at | 10398 | 2.72 | 1.86 | -1.45 | MYL9 | myosin, light chain 9, regulatory |
| **1** | 11754421_a_at | 23584 | 2.31 | 1.89 | -1.42 | VSIG2 | V-set and immunoglobulin domain containing 2 |
| **1** | 11754458_a_at | 84519 | 2.39 | 1.99 | -1.47 | ACRBP | acrosin binding protein |
| **1** | 11754824_a_at | 29094 | 2.02 | 2.04 | -1.31 | HSPC159 | galectin-related protein |
| **1** | 11754909_a_at | 84281 | 2.52 | 2.38 | -1.14 | C2orf88 | chromosome 2 open reading frame 88 |
| **1** | 11756275_a_at | 9124 | 2.10 | 1.96 | -1.28 | PDLIM1 | PDZ and LIM domain 1 |
| **1** | 11756547_a_at | 1191 | 3.12 | 2.06 | -1.76 | CLU | clusterin |
| **1** | 11757634_a_at | 3934 | 3.95 | 2.59 | 1.00 | LCN2 | lipocalin 2 |
| **1** | 11757651_x_at | 100128731 | 2.12 | 1.93 | -1.05 | OST4 | oligosaccharyltransferase 4 homolog (S. cerevisiae) |
| **1** | 11758987_at | 5196 | 2.57 | 2.36 | -1.40 | PF4 | platelet factor 4 |
| **1** | 11759005_a_at | 80763 | 2.26 | 2.29 | -1.37 | C12orf39 | chromosome 12 open reading frame 39 |
| **1** | 11759131_at | 8969 | 2.62 | 2.16 | 1.37 | HIST1H2AG | histone cluster 1, H2ag |
| **1** | 11759232_at | 80739 | 2.75 | 2.06 | -1.17 | C6orf25 | chromosome 6 open reading frame 25 |
| **1** | 11761349_x_at | 7168 | 2.28 | 1.78 | -1.12 | TPM1 | tropomyosin 1 (alpha) |
| **1** | 11763871_at | 6678 | 2.93 | 2.39 | -1.54 | SPARC | secreted protein, acidic, cysteine-rich (osteonectin) |
| **1** | 11763872_x_at | 6678 | 3.05 | 2.20 | -1.45 | SPARC | secreted protein, acidic, cysteine-rich (osteonectin) |
| **2** | 11726980_a_at | 79957 | 1.79 | 1.68 | 2.77 | PAQR6 | progestin and adipoQ receptor family member VI |
| **2** | 11728978_x_at | 841 | 1.28 | 1.49 | 2.09 | CASP8 | caspase 8, apoptosis-related cysteine peptidase |
| **2** | 11732275_at | 6352 | 1.93 | 2.03 | 1.64 | CCL5 | chemokine (C-C motif) ligand 5 |
| **2** | 11734843_x_at | 54737 | 1.85 | 2.07 | 1.94 | MPHOSPH8 | M-phase phosphoprotein 8 |
| **2** | 11734842_at | 54737 | 1.74 | 2.00 | 2.13 | MPHOSPH8 | M-phase phosphoprotein 8 |
| **2** | 11735845_x_at | 9720 | 1.05 | -1.04 | 2.09 | CCDC144A | coiled-coil domain containing 144A |
| **2** | 11735843_a_at | 9720 | -1.02 | -1.17 | 2.19 | CCDC144A | coiled-coil domain containing 144A |
| **2** | 11736979_at | 161835 | -1.57 | -1.36 | 2.29 | FSIP1 | fibrous sheath interacting protein 1 |
| **2** | 11737350_x_at | 442582 | 1.33 | 1.63 | 2.00 | STAG3L2 | stromal antigen 3-like 2 |
| **2** | 11742819_at | 5329 | 1.13 | -1.17 | 2.13 | PLAUR | plasminogen activator, urokinase receptor |
| **2** | 11745788_a_at | 10071 | -1.09 | -1.19 | 3.56 | MUC12 | mucin 12, cell surface associated |
| **2** | 11751380_x_at | 7439 | -1.06 | 1.02 | 2.72 | BEST1 | bestrophin 1 |
| **2** | 11752261_a_at | 7439 | -1.16 | -1.09 | 2.72 | BEST1 | bestrophin 1 |
| **2** | 11758924_x_at | 6648 | 2.00 | 1.56 | 2.21 | SOD2 | superoxide dismutase 2, mitochondrial |
| **2** | 11759165_s_at | 100288807 /// 100310812 /// 285955 /// 441251 /// 441273 /// 442572 /// 442590 /// 729545 /// 729597 | 1.13 | 1.21 | 2.35 | LOC100288807 /// LOC442572 /// LOC729545 /// SPDYE1 /// SPDYE2 /// SPDYE2L /// SPDYE5 /// SPDYE6 /// SPDYE7P | similar to Putative WBSCR19-like protein 7 /// WBSCR19-like protein 8-like /// WBSCR19-like protein 5-like /// speedy homolog E1 (Xenopus laevis) /// speedy homolog E2 (Xenopus laevis) /// WBSCR19-like protein 3 /// speedy homolog E5 (Xenopus laevis) /// speedy homolog E6 (Xenopus laevis) /// speedy homolog E7 (Xenopus laevis), pseudogene |
| **2** | 11759459_x_at | 100132406 /// 149013 /// 200030 /// 284565 /// 400818 /// 55672 /// 728841 /// 728912 /// 728936 | -1.29 | -1.41 | 2.41 | KIAA1245 /// LOC200030 /// NBPF1 /// NBPF10 /// NBPF11 /// NBPF15 /// NBPF16 /// NBPF8 /// NBPF9 | KIAA1245 /// neuroblastoma breakpoint family, member 11-like /// neuroblastoma breakpoint family, member 1 /// neuroblastoma breakpoint family, member 10 /// neuroblastoma breakpoint family, member 11 /// neuroblastoma breakpoint family, member 15 /// neuroblastoma breakpoint family, member 16 /// neuroblastoma breakpoint family, member 8 /// neuroblastoma breakpoint family, member 9 |
| **2** | 11759864_at | 23060 | 1.08 | 1.15 | 2.08 | ZNF609 | zinc finger protein 609 |
| **2** | 11761064_x_at | 441273 | 1.23 | 1.25 | 2.01 | SPDYE2 | Speedy homolog E2 (Xenopus laevis) |
| **2** | 11761320_at | 7439 | 1.36 | 1.35 | 2.97 | BEST1 | bestrophin 1 |
| **2** | 11761500_at | 1572 | -1.47 | -1.61 | 2.27 | CYP2F1 | cytochrome P450, family 2, subfamily F, polypeptide 1 |
| **2** | 11761815_at | 84230 | -1.04 | -1.01 | 2.04 | LRRC8C | leucine rich repeat containing 8 family, member C |
| **2** | 11761879_at | 51010 | 1.93 | 2.12 | 2.04 | EXOSC3 | exosome component 3 |
| **2** | 11762472_at |  | -1.21 | -1.08 | 2.04 |  |  |
| **2** | 11762729_at | 442092 | -1.11 | -1.20 | 2.00 | ARVP6125 | hypothetical LOC442092 |
| **2** | 11762939_at |  | -1.30 | -1.14 | 2.76 |  |  |
| **2** | 11764273_at |  | 1.05 | 1.22 | 2.01 |  |  |
| **3** | 11719121_a_at | 94274 | 2.01 | 1.87 | -1.17 | PPP1R14A | protein phosphatase 1, regulatory (inhibitor) subunit 14A |
| **3** | 11721553_a_at | 2550 | 2.03 | 1.74 | 1.52 | GABBR1 | gamma-aminobutyric acid (GABA) B receptor, 1 |
| **3** | 11721693_a_at | 1534 | 1.86 | 2.43 | 1.35 | CYB561 | cytochrome b-561 |
| **3** | 11721944_x_at | 11158 | 1.80 | 2.28 | -1.02 | RABL2B | RAB, member of RAS oncogene family-like 2B |
| **3** | 11722761_a_at | 2624 | 2.28 | 2.11 | 1.17 | GATA2 | GATA binding protein 2 |
| **3** | 11725211_a_at | 55344 | 2.20 | 1.96 | 1.14 | PLCXD1 | phosphatidylinositol-specific phospholipase C, X domain containing 1 |
| **3** | 11725677_a_at | 6810 | 1.80 | 2.02 | -1.06 | STX4 | syntaxin 4 |
| **3** | 11726557_a_at | 55876 | 2.09 | 2.39 | 1.27 | GSDMB | gasdermin B |
| **3** | 11727248_a_at | 4621 | 1.65 | 2.11 | 1.08 | MYH3 | myosin, heavy chain 3, skeletal muscle, embryonic |
| **3** | 11727473_at | 1178 | 2.35 | 2.39 | 1.15 | CLC | Charcot-Leyden crystal protein |
| **3** | 11729603_a_at | 4176 | 2.06 | 2.12 | 1.02 | MCM7 | minichromosome maintenance complex component 7 |
| **3** | 11729768_s_at | 8694 | 1.73 | 2.05 | 1.03 | DGAT1 | diacylglycerol O-acyltransferase homolog 1 (mouse) |
| **3** | 11729806_a_at | 91544 | 2.29 | 2.09 | 1.25 | UBXN11 | UBX domain protein 11 |
| **3** | 11730023_s_at | 29990 | 1.72 | 2.07 | 1.33 | PILRB | paired immunoglobin-like type 2 receptor beta |
| **3** | 11730837_x_at | 54853 | 2.05 | 2.31 | 1.50 | WDR55 | WD repeat domain 55 |
| **3** | 11734697_x_at | 23015 /// 440270 | 1.69 | 2.06 | 1.16 | GOLGA8A /// GOLGA8B | golgin A8 family, member A /// golgin A8 family, member B |
| **3** | 11734696_x_at | 100292334 /// 23015 /// 440270 | 1.72 | 2.17 | 1.14 | GOLGA8A /// GOLGA8B /// LOC100292334 | golgin A8 family, member A /// golgin A8 family, member B /// similar to GOLGA8B protein |
| **3** | 11735717_a_at | 7769 | 1.79 | 2.29 | 1.45 | ZNF226 | zinc finger protein 226 |
| **3** | 11737672_a_at | 80152 | 1.88 | 2.18 | -1.15 | CENPT | centromere protein T |
| **3** | 11740447_x_at | 11119 | 2.02 | 2.09 | 1.33 | BTN3A1 | butyrophilin, subfamily 3, member A1 |
| **3** | 11740744_x_at | 7169 | 1.51 | 2.12 | -1.22 | TPM2 | tropomyosin 2 (beta) |
| **3** | 11743475_a_at | 3687 | 2.50 | 2.09 | 1.28 | ITGAX | integrin, alpha X (complement component 3 receptor 4 subunit) |
| **3** | 11744157_s_at | 1120 /// 1375 /// 386593 | 2.20 | 2.22 | 1.11 | CHKB /// CHKB-CPT1B /// CPT1B | choline kinase beta /// choline kinase-like, carnitine palmitoyltransferase 1B (muscle) transcription unit /// carnitine palmitoyltransferase 1B (muscle) |
| **3** | 11744180_a_at | 10312 | 2.35 | 1.81 | -1.02 | TCIRG1 | T-cell, immune regulator 1, ATPase, H+ transporting, lysosomal V0 subunit A3 |
| **3** | 11744351_a_at | 22861 /// 728392 | 1.99 | 2.18 | 1.25 | LOC728392 /// NLRP1 | hypothetical protein LOC728392 /// NLR family, pyrin domain containing 1 |
| **3** | 11744408_x_at | 23015 | 1.79 | 2.29 | 1.14 | GOLGA8A | golgin A8 family, member A |
| **3** | 11744407_x_at | 23015 | 1.72 | 2.26 | 1.00 | GOLGA8A | golgin A8 family, member A |
| **3** | 11744440_s_at | 80279 | 1.94 | 2.06 | 1.05 | CDK5RAP3 | CDK5 regulatory subunit associated protein 3 |
| **3** | 11751846_a_at | 220064 | 1.51 | 2.23 | 1.65 | ORAOV1 | oral cancer overexpressed 1 |
| **3** | 11755785_a_at | 25956 | 2.13 | 2.20 | 1.16 | SEC31B | SEC31 homolog B (S. cerevisiae) |
| **3** | 11756427_x_at | 339231 | 1.84 | 2.10 | 1.06 | ARL16 | ADP-ribosylation factor-like 16 |
| **3** | 11757653_s_at | 80279 | 1.99 | 2.08 | 1.09 | CDK5RAP3 | CDK5 regulatory subunit associated protein 3 |
| **3** | 11759569_a_at | 80279 | 1.91 | 2.06 | 1.17 | CDK5RAP3 | CDK5 regulatory subunit associated protein 3 |
| **3** | 11759938_a_at | 55846 | 1.91 | 2.38 | 1.26 | ITFG2 | integrin alpha FG-GAP repeat containing 2 |
| **3** | 11760221_at | 837 | 2.07 | 1.87 | 1.41 | CASP4 | caspase 4, apoptosis-related cysteine peptidase |
| **3** | 11760413_x_at | 10980 | 1.67 | 2.02 | 1.21 | COPS6 | COP9 constitutive photomorphogenic homolog subunit 6 (Arabidopsis) |
| **3** | 11760872_at | 7132 | 2.07 | 1.76 | 1.28 | TNFRSF1A | tumor necrosis factor receptor superfamily, member 1A |
| **3** | 11761194_at | 10618 | 2.47 | 2.84 | 1.86 | TGOLN2 | trans-golgi network protein 2 |
| **3** | 11761195_x_at | 10618 | 2.25 | 2.39 | 1.37 | TGOLN2 | trans-golgi network protein 2 |
| **3** | 11761758_at | 5698 | 2.32 | 2.38 | 1.58 | PSMB9 | proteasome (prosome, macropain) subunit, beta type, 9 (large multifunctional peptidase 2) |
| **3** | 11762227_x_at | 27128 | 2.49 | 2.39 | 2.45 | CYTH4 | cytohesin 4 |
| **3** | 11762226_at | 27128 | 2.43 | 2.41 | 2.08 | CYTH4 | cytohesin 4 |
| **3** | 11763472_x_at | 340152 | 2.05 | 2.29 | 1.06 | ZC3H12D | zinc finger CCCH-type containing 12D |
| **3** | 11763755_a_at | 10578 | 1.71 | 2.38 | 1.55 | GNLY | granulysin |
| **3** | 11763756_x_at | 10578 | 1.64 | 2.05 | 1.43 | GNLY | granulysin |
| **4** | 11715132_x_at | 8345 | 2.06 | 1.67 | -1.08 | HIST1H2BH | histone cluster 1, H2bh |
| **4** | 11716523_at | 6280 | 2.10 | 1.17 | -1.20 | S100A9 | S100 calcium binding protein A9 |
| **4** | 11716710_a_at | 133 | 2.46 | 1.16 | -1.16 | ADM | adrenomedullin |
| **4** | 11718031_a_at | 535 | 2.11 | 1.61 | 1.29 | ATP6V0A1 | ATPase, H+ transporting, lysosomal V0 subunit a1 |
| **4** | 11718058_a_at | 7298 | 2.54 | 1.36 | -1.08 | TYMS | thymidylate synthetase |
| **4** | 11718183_at | 113130 | 2.06 | 1.20 | 1.13 | CDCA5 | cell division cycle associated 5 |
| **4** | 11718228_a_at | 91300 | 2.03 | 1.64 | -1.00 | C19orf22 | chromosome 19 open reading frame 22 |
| **4** | 11718347_a_at | 6286 | 2.06 | 1.51 | 1.15 | S100P | S100 calcium binding protein P |
| **4** | 11718350_s_at | 55655 | 2.03 | 1.30 | 1.04 | NLRP2 | NLR family, pyrin domain containing 2 |
| **4** | 11720007_a_at | 79689 | 2.34 | 1.26 | 1.24 | STEAP4 | STEAP family member 4 |
| **4** | 11720608_a_at | 6285 | -1.25 | -1.20 | -3.12 | S100B | S100 calcium binding protein B |
| **4** | 11720970_at | 7153 | 2.12 | 1.06 | -1.26 | TOP2A | topoisomerase (DNA) II alpha 170kDa |
| **4** | 11721143_a_at | 4288 | 2.28 | 1.11 | 1.00 | MKI67 | antigen identified by monoclonal antibody Ki-67 |
| **4** | 11721438_s_at | 1667 /// 1668 /// 728358 | 5.21 | 3.73 | 3.25 | DEFA1 /// DEFA1B /// DEFA3 | defensin, alpha 1 /// defensin, alpha 1B /// defensin, alpha 3, neutrophil-specific |
| **4** | 11721499_x_at | 5476 | 2.05 | 1.37 | -1.36 | CTSA | cathepsin A |
| **4** | 11722558_x_at | 4354 | 2.41 | 1.70 | -1.02 | MPP1 | membrane protein, palmitoylated 1, 55kDa |
| **4** | 11722724_a_at | 1439 | 2.22 | 1.69 | 1.14 | CSF2RB | colony stimulating factor 2 receptor, beta, low-affinity (granulocyte-macrophage) |
| **4** | 11723050_a_at | 3310 | 2.59 | 1.84 | 1.22 | HSPA6 | heat shock 70kDa protein 6 (HSP70B') |
| **4** | 11723881_a_at | 4778 | 2.15 | 1.55 | 1.15 | NFE2 | nuclear factor (erythroid-derived 2), 45kDa |
| **4** | 11724089_a_at | 53917 | 2.13 | 1.81 | 1.24 | RAB24 | RAB24, member RAS oncogene family |
| **4** | 11726032_a_at | 1456 | -1.94 | -1.80 | -2.14 | CSNK1G3 | casein kinase 1, gamma 3 |
| **4** | 11726033_x_at | 1456 | -2.06 | -1.91 | -2.39 | CSNK1G3 | casein kinase 1, gamma 3 |
| **4** | 11726328_x_at | 2633 | 2.67 | 1.85 | 1.72 | GBP1 | guanylate binding protein 1, interferon-inducible, 67kDa |
| **4** | 11726329_x_at | 2633 | 2.29 | 1.49 | 1.26 | GBP1 | guanylate binding protein 1, interferon-inducible, 67kDa |
| **4** | 11726616_a_at | 8685 | 2.18 | 1.56 | 1.06 | MARCO | macrophage receptor with collagenous structure |
| **4** | 11728267_s_at | 10288 /// 10859 | 2.16 | 1.36 | 1.33 | LILRB1 /// LILRB2 | leukocyte immunoglobulin-like receptor, subfamily B (with TM and ITIM domains), member 1 /// leukocyte immunoglobulin-like receptor, subfamily B (with TM and ITIM domains), member 2 |
| **4** | 11729140_x_at | 57823 | 2.15 | 1.80 | 1.23 | SLAMF7 | SLAM family member 7 |
| **4** | 11729138_a_at | 57823 | 2.08 | 1.68 | 1.24 | SLAMF7 | SLAM family member 7 |
| **4** | 11729291_a_at | 51312 | 2.14 | 1.38 | -1.05 | SLC25A37 | solute carrier family 25, member 37 |
| **4** | 11729382_a_at | 26289 | -1.42 | -1.25 | -2.80 | AK5 | adenylate kinase 5 |
| **4** | 11730457_a_at | 9447 | 2.67 | 2.19 | 1.42 | AIM2 | absent in melanoma 2 |
| **4** | 11730458_at | 9447 | 2.32 | 1.94 | 1.15 | AIM2 | absent in melanoma 2 |
| **4** | 11730466_a_at | 3759 | 2.38 | 1.26 | 1.23 | KCNJ2 | potassium inwardly-rectifying channel, subfamily J, member 2 |
| **4** | 11730847_a_at | 695 | 2.47 | 2.08 | 1.36 | BTK | Bruton agammaglobulinemia tyrosine kinase |
| **4** | 11731425_at | 3579 | 2.26 | 1.77 | 1.03 | CXCR2 | chemokine (C-X-C motif) receptor 2 |
| **4** | 11731426_s_at | 3579 /// 3580 | 2.72 | 1.87 | -1.12 | CXCR2 /// CXCR2P1 | chemokine (C-X-C motif) receptor 2 /// chemokine (C-X-C motif) receptor 2 pseudogene 1 |
| **4** | 11732265_at | 10859 | 2.27 | 1.70 | 1.28 | LILRB1 | leukocyte immunoglobulin-like receptor, subfamily B (with TM and ITIM domains), member 1 |
| **4** | 11732266_x_at | 10859 | 2.07 | 1.60 | 1.08 | LILRB1 | leukocyte immunoglobulin-like receptor, subfamily B (with TM and ITIM domains), member 1 |
| **4** | 11732425_at | 118932 | 2.12 | 1.27 | 1.45 | ANKRD22 | ankyrin repeat domain 22 |
| **4** | 11732454_at | 3772 | 2.04 | 1.35 | 1.07 | KCNJ15 | potassium inwardly-rectifying channel, subfamily J, member 15 |
| **4** | 11733005_at | 2215 | 3.86 | 2.67 | -1.07 | FCGR3B | Fc fragment of IgG, low affinity IIIb, receptor (CD16b) |
| **4** | 11733439_a_at | 115362 | 2.09 | 1.75 | 1.10 | GBP5 | guanylate binding protein 5 |
| **4** | 11733740_at | 8342 | 2.46 | 1.38 | -1.15 | HIST1H2BM | histone cluster 1, H2bm |
| **4** | 11734035_a_at | 2358 | 2.11 | 1.14 | 1.70 | FPR2 | formyl peptide receptor 2 |
| **4** | 11734201_s_at | 2710 | 2.16 | 1.19 | 1.07 | GK | glycerol kinase |
| **4** | 11736311_x_at | 2209 | 3.34 | 1.38 | 1.25 | FCGR1A | Fc fragment of IgG, high affinity Ia, receptor (CD64) |
| **4** | 11737388_s_at | 10903 | 2.41 | 1.89 | 1.48 | MTMR11 | myotubularin related protein 11 |
| **4** | 11738208_a_at | 212 | 2.28 | 1.34 | 1.03 | ALAS2 | aminolevulinate, delta-, synthase 2 |
| **4** | 11739140_a_at | 2358 | 2.06 | 1.04 | 1.42 | FPR2 | formyl peptide receptor 2 |
| **4** | 11739552_a_at | 9586 | 2.07 | 1.11 | 1.22 | CREB5 | cAMP responsive element binding protein 5 |
| **4** | 11742890_at | 84057 | 2.11 | 1.25 | -1.07 | MND1 | meiotic nuclear divisions 1 homolog (S. cerevisiae) |
| **4** | 11742988_s_at | 7430 | -1.19 | -1.05 | -2.14 | EZR | ezrin |
| **4** | 11743065_at | 990 | 2.03 | 1.26 | 1.20 | CDC6 | cell division cycle 6 homolog (S. cerevisiae) |
| **4** | 11743730_at | 8743 | 2.90 | 1.87 | 1.30 | TNFSF10 | tumor necrosis factor (ligand) superfamily, member 10 |
| **4** | 11743731_a_at | 8743 | 2.01 | 1.45 | 1.20 | TNFSF10 | tumor necrosis factor (ligand) superfamily, member 10 |
| **4** | 11744313_a_at | 400410 | 2.08 | 1.95 | -1.14 | ST20 | suppressor of tumorigenicity 20 |
| **4** | 11744426_x_at | 9768 | 2.06 | 1.28 | -1.13 | KIAA0101 | KIAA0101 |
| **4** | 11744434_a_at | 83666 | 2.35 | 1.44 | 1.52 | PARP9 | poly (ADP-ribose) polymerase family, member 9 |
| **4** | 11744559_a_at | 51312 | 2.54 | 1.59 | -1.17 | SLC25A37 | solute carrier family 25, member 37 |
| **4** | 11757277_x_at | 3240 | 2.68 | 1.32 | 1.07 | HP | haptoglobin |
| **4** | 11745504_a_at | 4354 | 2.52 | 1.67 | -1.02 | MPP1 | membrane protein, palmitoylated 1, 55kDa |
| **4** | 11745903_a_at | 57823 | 2.11 | 1.69 | 1.28 | SLAMF7 | SLAM family member 7 |
| **4** | 11746013_s_at | 653361 /// 654816 /// 654817 | 2.21 | 1.77 | 1.39 | NCF1 /// NCF1B /// NCF1C | neutrophil cytosolic factor 1 /// neutrophil cytosolic factor 1B pseudogene /// neutrophil cytosolic factor 1C pseudogene |
| **4** | 11746894_x_at | 4354 | 2.33 | 1.61 | 1.06 | MPP1 | membrane protein, palmitoylated 1, 55kDa |
| **4** | 11746893_a_at | 4354 | 2.23 | 1.53 | 1.06 | MPP1 | membrane protein, palmitoylated 1, 55kDa |
| **4** | 11747803_a_at | 1196 | 2.01 | 1.83 | 1.14 | CLK2 | CDC-like kinase 2 |
| **4** | 11747952_x_at | 8743 | 2.64 | 1.88 | 1.57 | TNFSF10 | tumor necrosis factor (ligand) superfamily, member 10 |
| **4** | 11748622_s_at | 5476 | 2.27 | 1.49 | -1.10 | CTSA | cathepsin A |
| **4** | 11749773_x_at | 2633 | 2.00 | 1.40 | 1.17 | GBP1 | guanylate binding protein 1, interferon-inducible, 67kDa |
| **4** | 11750149_a_at | 401303 | -1.38 | -1.01 | -2.21 | ZNF815 | zinc finger protein 815 |
| **4** | 11750879_a_at | 9586 | 2.08 | 1.14 | 1.37 | CREB5 | cAMP responsive element binding protein 5 |
| **4** | 11751116_a_at | 87 | 1.08 | -1.06 | -2.09 | ACTN1 | actinin, alpha 1 |
| **4** | 11751117_x_at | 87 | 1.01 | -1.08 | -2.01 | ACTN1 | actinin, alpha 1 |
| **4** | 11751600_a_at | 27071 | 2.01 | 1.81 | -1.14 | DAPP1 | dual adaptor of phosphotyrosine and 3-phosphoinositides |
| **4** | 11751611_a_at | 10906 | 2.11 | 1.77 | -1.14 | TRAFD1 | TRAF-type zinc finger domain containing 1 |
| **4** | 11751805_a_at | 7298 | 2.26 | 1.47 | -1.03 | TYMS | thymidylate synthetase |
| **4** | 11752930_a_at | 2633 | 2.91 | 1.85 | 1.64 | GBP1 | guanylate binding protein 1, interferon-inducible, 67kDa |
| **4** | 11752931_x_at | 2633 | 2.45 | 1.69 | 1.32 | GBP1 | guanylate binding protein 1, interferon-inducible, 67kDa |
| **4** | 11753179_s_at | 54463 | -1.53 | -1.35 | -2.03 | FAM134B | family with sequence similarity 134, member B |
| **4** | 11753429_a_at | 23558 | 2.10 | 1.74 | 1.16 | WBP2 | WW domain binding protein 2 |
| **4** | 11754115_a_at | 6285 | -1.46 | -1.15 | -2.52 | S100B | S100 calcium binding protein B |
| **4** | 11754360_a_at | 6241 | 3.10 | 1.34 | -1.40 | RRM2 | ribonucleotide reductase M2 |
| **4** | 11755688_a_at | 54507 | 1.64 | 1.50 | 2.34 | ADAMTSL4 | ADAMTS-like 4 |
| **4** | 11756309_x_at | 53917 | 2.06 | 1.81 | 1.14 | RAB24 | RAB24, member RAS oncogene family |
| **4** | 11756334_x_at | 306 | 2.06 | 1.55 | -1.10 | ANXA3 | annexin A3 |
| **4** | 11756362_x_at | 2210 | 2.70 | 1.23 | -1.06 | FCGR1B | Fc fragment of IgG, high affinity Ib, receptor (CD64) |
| **4** | 11756406_x_at | 133 | 2.26 | 1.11 | -1.23 | ADM | adrenomedullin |
| **4** | 11756626_s_at | 11130 | 2.58 | 1.61 | 1.21 | ZWINT | ZW10 interactor |
| **4** | 11744649_x_at | 3240 | 2.03 | 1.17 | 1.03 | HP | haptoglobin |
| **4** | 11757367_s_at | 3310 /// 3311 | 2.76 | 2.12 | 1.25 | HSPA6 /// HSPA7 | heat shock 70kDa protein 6 (HSP70B') /// heat shock 70kDa protein 7 (HSP70B) |
| **4** | 11758219_x_at | 6241 | 3.03 | 1.25 | -1.14 | RRM2 | ribonucleotide reductase M2 |
| **4** | 11758412_s_at | 212 | 2.60 | 1.48 | 1.11 | ALAS2 | aminolevulinate, delta-, synthase 2 |
| **4** | 11759111_x_at | 8345 | 2.28 | 1.65 | -1.00 | HIST1H2BH | histone cluster 1, H2bh |
| **4** | 11759110_a_at | 8345 | 2.22 | 1.42 | -1.14 | HIST1H2BH | histone cluster 1, H2bh |
| **4** | 11759237_at | 64581 | 1.36 | 1.11 | 2.28 | CLEC7A | C-type lectin domain family 7, member A |
| **4** | 11759238_x_at | 64581 | 1.33 | 1.06 | 2.17 | CLEC7A | C-type lectin domain family 7, member A |
| **4** | 11759560_s_at | 51312 | 2.37 | 1.73 | -1.01 | SLC25A37 | solute carrier family 25, member 37 |
| **4** | 11759618_a_at | 51312 | 2.26 | 1.41 | 1.14 | SLC25A37 | solute carrier family 25, member 37 |
| **4** | 11759559_at | 51312 | 2.68 | 1.88 | -1.29 | SLC25A37 | solute carrier family 25, member 37 |
| **4** | 11759815_a_at | 3507 | 2.84 | 2.05 | -1.71 | IGHM | immunoglobulin heavy constant mu |
| **4** | 11759816_x_at | 3507 | 2.34 | 1.61 | -1.76 | IGHM | immunoglobulin heavy constant mu |
| **4** | 11762101_at | 160364 | 2.09 | 1.66 | 1.63 | CLEC12A | C-type lectin domain family 12, member A |
| **4** | 11764046_x_at | 3507 | 2.46 | 1.82 | -1.72 | IGHM | immunoglobulin heavy constant mu |
| **5** | 11716631_s_at | 5055 | -2.56 | -3.86 | -1.34 | SERPINB2 | serpin peptidase inhibitor, clade B (ovalbumin), member 2 |
| **5** | 11717732_s_at | 5209 | -1.57 | -2.34 | 1.22 | PFKFB3 | 6-phosphofructo-2-kinase/fructose-2,6-biphosphatase 3 |
| **5** | 11717860_a_at | 1958 | -4.01 | -3.23 | 1.19 | EGR1 | early growth response 1 |
| **5** | 11717861_a_at | 1958 | -1.94 | -2.14 | -1.05 | EGR1 | early growth response 1 |
| **5** | 11717862_x_at | 1958 | -2.00 | -2.00 | 1.09 | EGR1 | early growth response 1 |
| **5** | 11718012_at | 8660 | -1.89 | -2.36 | 1.02 | IRS2 | insulin receptor substrate 2 |
| **5** | 11718013_at | 8660 | -2.17 | -2.93 | -1.53 | IRS2 | insulin receptor substrate 2 |
| **5** | 11718394_at | 3725 | -2.11 | -2.09 | -1.71 | JUN | jun oncogene |
| **5** | 11718758_a_at | 3091 | -1.45 | -2.08 | 1.35 | HIF1A | hypoxia inducible factor 1, alpha subunit (basic helix-loop-helix transcription factor) |
| **5** | 11718841_s_at | 3576 | -4.20 | -4.37 | 1.78 | IL8 | interleukin 8 |
| **5** | 11718939_s_at | 7128 | -2.74 | -2.57 | 1.24 | TNFAIP3 | tumor necrosis factor, alpha-induced protein 3 |
| **5** | 11718940_a_at | 7128 | -2.85 | -2.45 | -1.07 | TNFAIP3 | tumor necrosis factor, alpha-induced protein 3 |
| **5** | 11719898_s_at | 1839 | -3.34 | -3.93 | 1.21 | HBEGF | heparin-binding EGF-like growth factor |
| **5** | 11719916_at | 3553 | -1.18 | -1.69 | 3.60 | IL1B | interleukin 1, beta |
| **5** | 11720409_s_at | 1545 | -1.52 | -2.08 | 1.08 | CYP1B1 | cytochrome P450, family 1, subfamily B, polypeptide 1 |
| **5** | 11720410_s_at | 1545 | -1.47 | -2.20 | -1.04 | CYP1B1 | cytochrome P450, family 1, subfamily B, polypeptide 1 |
| **5** | 11720411_s_at | 1545 | -1.55 | -2.28 | -1.04 | CYP1B1 | cytochrome P450, family 1, subfamily B, polypeptide 1 |
| **5** | 11721093_a_at | 7057 | -1.50 | -2.17 | 1.36 | THBS1 | thrombospondin 1 |
| **5** | 11721092_a_at | 7057 | -2.24 | -2.92 | 1.01 | THBS1 | thrombospondin 1 |
| **5** | 11721091_a_at | 7057 | -2.72 | -3.35 | 1.28 | THBS1 | thrombospondin 1 |
| **5** | 11721090_at | 7057 | -4.54 | -6.62 | 1.44 | THBS1 | thrombospondin 1 |
| **5** | 11722015_at | 4973 | -1.26 | -1.47 | 2.42 | OLR1 | oxidized low density lipoprotein (lectin-like) receptor 1 |
| **5** | 11722016_a_at | 4973 | -1.40 | -1.38 | 2.91 | OLR1 | oxidized low density lipoprotein (lectin-like) receptor 1 |
| **5** | 11722017_x_at | 4973 | -1.48 | -1.55 | 2.37 | OLR1 | oxidized low density lipoprotein (lectin-like) receptor 1 |
| **5** | 11722728_a_at | 1959 | -2.42 | -1.92 | -1.13 | EGR2 | early growth response 2 |
| **5** | 11723679_s_at | 969 | -2.67 | -2.50 | -1.04 | CD69 | CD69 molecule |
| **5** | 11724037_at | 5743 | -1.74 | -2.14 | 2.16 | PTGS2 | prostaglandin-endoperoxide synthase 2 (prostaglandin G/H synthase and cyclooxygenase) |
| **5** | 11725631_a_at | 4929 | -2.56 | -2.27 | 1.19 | NR4A2 | nuclear receptor subfamily 4, group A, member 2 |
| **5** | 11747474_a_at | 4929 | -3.39 | -2.38 | 1.14 | NR4A2 | nuclear receptor subfamily 4, group A, member 2 |
| **5** | 11727428_at | 1880 | -2.37 | -1.97 | -1.31 | GPR183 | G protein-coupled receptor 183 |
| **5** | 11727757_at | 5008 | -1.80 | -2.27 | -1.27 | OSM | oncostatin M |
| **5** | 11727779_at | 27069 | -2.20 | -2.05 | -1.31 | GHITM | growth hormone inducible transmembrane protein |
| **5** | 11728191_x_at | 7852 | -2.08 | -1.97 | 1.01 | CXCR4 | chemokine (C-X-C motif) receptor 4 |
| **5** | 11728189_a_at | 7852 | -2.08 | -2.00 | 1.01 | CXCR4 | chemokine (C-X-C motif) receptor 4 |
| **5** | 11728477_at | 2921 | 1.20 | 1.03 | 2.28 | CXCL3 | chemokine (C-X-C motif) ligand 3 |
| **5** | 11731027_s_at | 1390 | -2.15 | -2.33 | -1.24 | CREM | cAMP responsive element modulator |
| **5** | 11732277_a_at | 27086 | -2.07 | -1.75 | -1.33 | FOXP1 | forkhead box P1 |
| **5** | 11732718_at | 2069 | -1.65 | -1.95 | 2.49 | EREG | epiregulin |
| **5** | 11732720_a_at | 2069 | -1.69 | -2.37 | 3.16 | EREG | epiregulin |
| **5** | 11733022_at | 694 | -2.07 | -2.07 | 1.05 | BTG1 | B-cell translocation gene 1, anti-proliferative |
| **5** | 11733593_a_at | 5858 | -2.03 | -1.70 | 2.11 | PZP | pregnancy-zone protein |
| **5** | 11734712_a_at | 5142 | -1.72 | -2.15 | -1.26 | PDE4B | phosphodiesterase 4B, cAMP-specific (phosphodiesterase E4 dunce homolog, Drosophila) |
| **5** | 11735219_at | 1300 | -2.03 | -1.65 | 1.13 | COL10A1 | collagen, type X, alpha 1 |
| **5** | 11736920_a_at | 65986 | -2.17 | -1.97 | -1.48 | ZBTB10 | zinc finger and BTB domain containing 10 |
| **5** | 11738577_at | 127623 | -1.11 | -1.24 | 3.01 | OR2B11 | olfactory receptor, family 2, subfamily B, member 11 |
| **5** | 11739094_a_at | 7852 | -2.10 | -1.98 | -1.11 | CXCR4 | chemokine (C-X-C motif) receptor 4 |
| **5** | 11740030_s_at | 1390 | -2.03 | -2.12 | -1.40 | CREM | cAMP responsive element modulator |
| **5** | 11740028_a_at | 1390 | -2.10 | -2.07 | -1.26 | CREM | cAMP responsive element modulator |
| **5** | 11741321_a_at | 1390 | -2.19 | -1.98 | -1.08 | CREM | cAMP responsive element modulator |
| **5** | 11741079_x_at | 1390 | -2.60 | -2.34 | -1.19 | CREM | cAMP responsive element modulator |
| **5** | 11741705_at | 8013 | -2.31 | -2.28 | 1.07 | NR4A3 | nuclear receptor subfamily 4, group A, member 3 |
| **5** | 11742765_at | 5996 | -2.94 | -2.71 | 1.12 | RGS1 | regulator of G-protein signaling 1 |
| **5** | 11743000_at | 9308 | -2.67 | -2.51 | 1.28 | CD83 | CD83 molecule |
| **5** | 11743110_at | 10135 | -1.76 | -2.89 | 1.60 | NAMPT | nicotinamide phosphoribosyltransferase |
| **5** | 11743972_a_at | 54541 | -2.07 | -2.16 | 1.03 | DDIT4 | DNA-damage-inducible transcript 4 |
| **5** | 11744128_x_at | 2920 | -1.25 | -1.66 | 2.58 | CXCL2 | chemokine (C-X-C motif) ligand 2 |
| **5** | 11744127_at | 2920 | -1.41 | -1.90 | 2.98 | CXCL2 | chemokine (C-X-C motif) ligand 2 |
| **5** | 11744219_at | 50486 | -3.10 | -3.77 | 1.02 | G0S2 | G0/G1switch 2 |
| **5** | 11746907_a_at | 5144 | -1.77 | -2.03 | -1.40 | PDE4D | phosphodiesterase 4D, cAMP-specific (phosphodiesterase E3 dunce homolog, Drosophila) |
| **5** | 11747104_s_at | 1545 | -1.49 | -2.37 | -1.41 | CYP1B1 | cytochrome P450, family 1, subfamily B, polypeptide 1 |
| **5** | 11725632_at | 4929 | -2.23 | -1.77 | 1.04 | NR4A2 | nuclear receptor subfamily 4, group A, member 2 |
| **5** | 11748146_at | 969 | -1.69 | -2.08 | -1.08 | CD69 | CD69 molecule |
| **5** | 11750513_a_at | 4973 | -1.41 | -1.44 | 2.35 | OLR1 | oxidized low density lipoprotein (lectin-like) receptor 1 |
| **5** | 11751197_a_at | 5858 | -2.34 | -1.83 | 1.78 | PZP | pregnancy-zone protein |
| **5** | 11751643_x_at | 1958 | -2.45 | -2.06 | -1.11 | EGR1 | early growth response 1 |
| **5** | 11752940_a_at | 1958 | -3.37 | -3.12 | 1.19 | EGR1 | early growth response 1 |
| **5** | 11753498_at | 643036 | -1.73 | -2.76 | 1.39 | SLED1 | RTFV9368 |
| **5** | 11754026_a_at | 3576 | -2.69 | -3.39 | 1.80 | IL8 | interleukin 8 |
| **5** | 11754334_s_at | 1958 | -2.83 | -2.26 | 1.03 | EGR1 | early growth response 1 |
| **5** | 11755700_a_at | 25976 | -2.21 | -2.04 | -1.28 | TIPARP | TCDD-inducible poly(ADP-ribose) polymerase |
| **5** | 11755850_a_at | 65986 | -2.42 | -1.85 | -1.48 | ZBTB10 | zinc finger and BTB domain containing 10 |
| **5** | 11755981_a_at | 1839 | -2.58 | -2.99 | 1.09 | HBEGF | heparin-binding EGF-like growth factor |
| **5** | 11758555_s_at | 1880 | -2.58 | -2.07 | -1.17 | GPR183 | G protein-coupled receptor 183 |
| **5** | 11758668_s_at | 100294341 /// 51326 | -1.93 | -2.08 | -1.08 | ARL17A /// LOC100294341 | ADP-ribosylation factor-like 17A /// similar to ADP-ribosylation factor-like 17 |
| **5** | 11758842_at | 7057 | -5.64 | -7.00 | 1.74 | THBS1 | Thrombospondin 1 |
| **5** | 11759423_at | 83481 | -2.94 | -2.44 | -1.22 | EPPK1 | epiplakin 1 |
| **5** | 11760293_at | 6648 | 1.01 | -1.18 | 2.08 | SOD2 | superoxide dismutase 2, mitochondrial |
| **5** | 11760294_a_at | 1604 | -1.93 | -2.32 | 1.27 | CD55 | CD55 molecule, decay accelerating factor for complement (Cromer blood group) |
| **5** | 11760312_at | 10135 | -2.75 | -3.98 | 1.84 | NAMPT | nicotinamide phosphoribosyltransferase |
| **5** | 11760549_at | 5500 | -3.02 | -2.71 | -1.04 | PPP1CB | protein phosphatase 1, catalytic subunit, beta isozyme |
| **5** | 11760874_at | 57602 | -1.99 | -2.03 | -1.02 | USP36 | ubiquitin specific peptidase 36 |
| **5** | 11761557_at | 51202 | -2.13 | -1.66 | 1.11 | DDX47 | DEAD (Asp-Glu-Ala-Asp) box polypeptide 47 |
| **5** | 11761890_at |  | -1.62 | -1.40 | 2.61 |  |  |
| **5** | 11762538_at | 10396 | -2.06 | -1.84 | -1.31 | ATP8A1 | ATPase, aminophospholipid transporter (APLT), class I, type 8A, member 1 |
| **5** | 11763226_x_at | 3576 | -2.09 | -2.62 | 1.56 | IL8 | interleukin 8 |

^a^ Values shown are fold change relative to healthy donors
